# Pinene-Based Oxidative Synthetic Toolbox for Scalable Polyester Synthesis

**DOI:** 10.1021/jacsau.1c00312

**Published:** 2021-10-08

**Authors:** Arne Stamm, Johannes Öhlin, Caroline Mosbech, Peter Olsén, Boyang Guo, Elisabeth Söderberg, Antonino Biundo, Linda Fogelström, Shubhankar Bhattacharyya, Uwe T. Bornscheuer, Eva Malmström, Per-Olof Syrén

**Affiliations:** † School of Engineering Sciences in Chemistry, Biotechnology and Health, Department of Fibre and Polymer Technology, Division of Coating Technology, KTH Royal Institute of Technology, Teknikringen 56-58, SE-100 44 Stockholm, Sweden; ‡ School of Engineering Sciences in Chemistry, Biotechnology and Health, Science for Life Laboratory, KTH Royal Institute of Technology, Tomtebodavägen 23, Box 1031, SE-171 21 Solna, Sweden; § School of Engineering Sciences in Chemistry, Biotechnology and Health, Department of Fibre and Polymer Technology, Wallenberg Wood Science Center, KTH Royal Institute of Technology, Teknikringen 56-58, Stockholm SE-100 44 Sweden; ∥ RISE Processum AB, Hörneborgsvägen 10, Domsjö, Örnsköldsvik 89258, Sweden; ⊥ Department of Biotechnology and Enzyme Catalysis, University of Greifswald, Institute of Biochemistry, Felix-Hausdorff-Strasse 4, 17487 Greifswald, Germany

**Keywords:** biobased polymers, coatings, terpenes, α-pinene, terpene lactone, diol

## Abstract

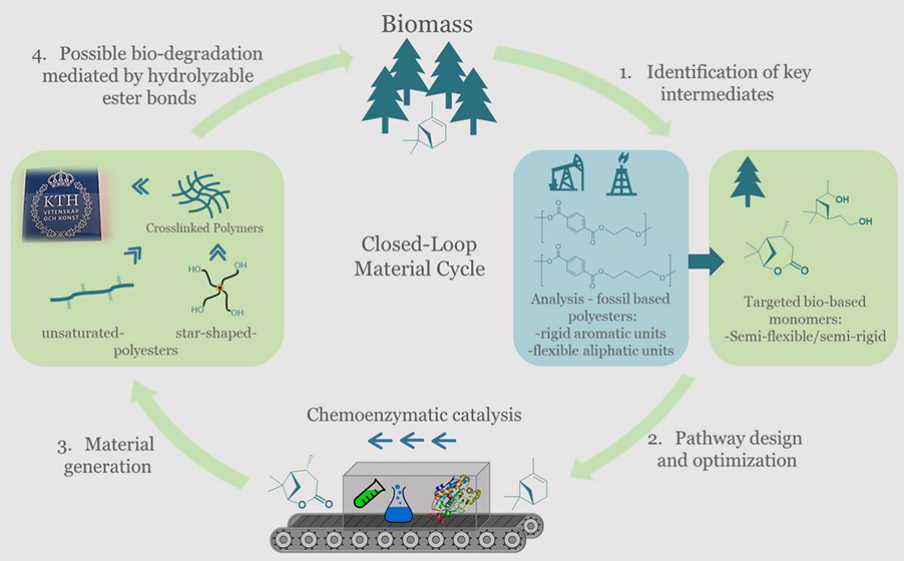

Generation of renewable polymers is a long-standing goal toward reaching a more sustainable society, but building blocks in biomass can be incompatible with desired polymerization type, hampering the full implementation potential of biomaterials. Herein, we show how conceptually simple oxidative transformations can be used to unlock the inherent reactivity of terpene synthons in generating polyesters by two different mechanisms starting from the same α-pinene substrate. In the first pathway, α-pinene was oxidized into the bicyclic verbanone-based lactone and subsequently polymerized into star-shaped polymers via ring-opening polymerization, resulting in a biobased semicrystalline polyester with tunable glass transition and melting temperatures. In a second pathway, polyesters were synthesized via polycondensation, utilizing the diol 1-(1′-hydroxyethyl)-3-(2′-hydroxy-ethyl)-2,2-dimethylcyclobutane (HHDC) synthesized by oxidative cleavage of the double bond of α-pinene, together with unsaturated biobased diesters such as dimethyl maleate (DMM) and dimethyl itaconate (DMI). The resulting families of terpene-based polyesters were thereafter successfully cross-linked by either transetherification, utilizing the terminal hydroxyl groups of the synthesized verbanone-based materials, or by UV irradiation, utilizing the unsaturation provided by the DMM or DMI moieties within the HHDC-based copolymers. This work highlights the potential to apply an oxidative toolbox to valorize inert terpene metabolites enabling generation of biosourced polyesters and coatings thereof by complementary mechanisms.

## Introduction

The current synthetic polymer economy implemented in society is not sustainable: the bulk (∼98%)^1^ of all man-made materials are fossil-based, and their production is associated with resource inefficiency,^2^ accumulation of nonrecyclable plastic waste, and severe environmental impacts.^3^ Each year, more than 350 mega tons (Mt) of petroleum-based plastics is currently synthesized,^4^ a number projected to triple within the next few decades,^5,6^ leading to the increase of CO_2_ emissions that contribute to climate change.^7^ Innovative technologies for the generation of closed-loop sustainable materials from renewable, non-food-based feedstocks are urgently needed,^8,9^ a development obstructed by bottlenecks that include the following: (i) challenging (bio)synthetic routes to reproduce fossil-based monomers,^10^ (ii) inert backbones of building blocks in biomass preventing desired polymerization under mild conditions, and (iii) lack of recycling technologies to valorize postconsumer materials back to monomers to enable a new life cycle that is not dependent on virgin synthons.^11^ One potent strategy that potentially tackles the above-mentioned points consists of divergent generation of biobased polymers harboring difference in structure, but with chemical and physical properties similar to currently industrially implemented synthetic polymers.^9,12^

Direct substitution of fossil-based counterparts by underutilized side streams, in particular, hemicellulose, lignin, and terpenes,^13^ is appealing but requires extensive transformations to replicate a small set of petroleum-based structures.^14^ Utilizing potentially valuable natural products, such as secondary metabolites abundant in side streams, as a nutrient source in fermentation would seem chemically counterintuitive, as this strategy would involve breaking down molecules already invested in by Nature during biosynthesis. Growing selected crops for bioplastic production is dependent on the use of arable land and the use of pesticides and fertilizers.^15^ What if we would instead adapt synthesis routes to fit each particular biomass-derived substrate according to a set of conceptually simple, broadly applicable chemical transformations? The importance of the latter strategy for facilitated access to biobased products has recently been emphasized in the literature.^14^

The fundamental importance of oxidative transformations is widely acknowledged in natural product chemistry^16^ and biomass valorization.^17^ We reasoned that oxidative chemistries could lay the foundation for the production of biosourced polyesters with both flexible aliphatic- and rigid aromatic-type of motifs that could be combined appropriately to approach properties of currently implemented materials. Polyesters are of great interest due to their properties and their possible (bio)degradation imposed by the hydrolyzable ester bonds.^9,18,19^ We hypothesize that incorporation of cyclic motifs allows tailor-made production of semirigid/flexible polyesters with appropriate thermal properties^20^ and that terpenes are a key source for their preparation.

**Scheme 1:**
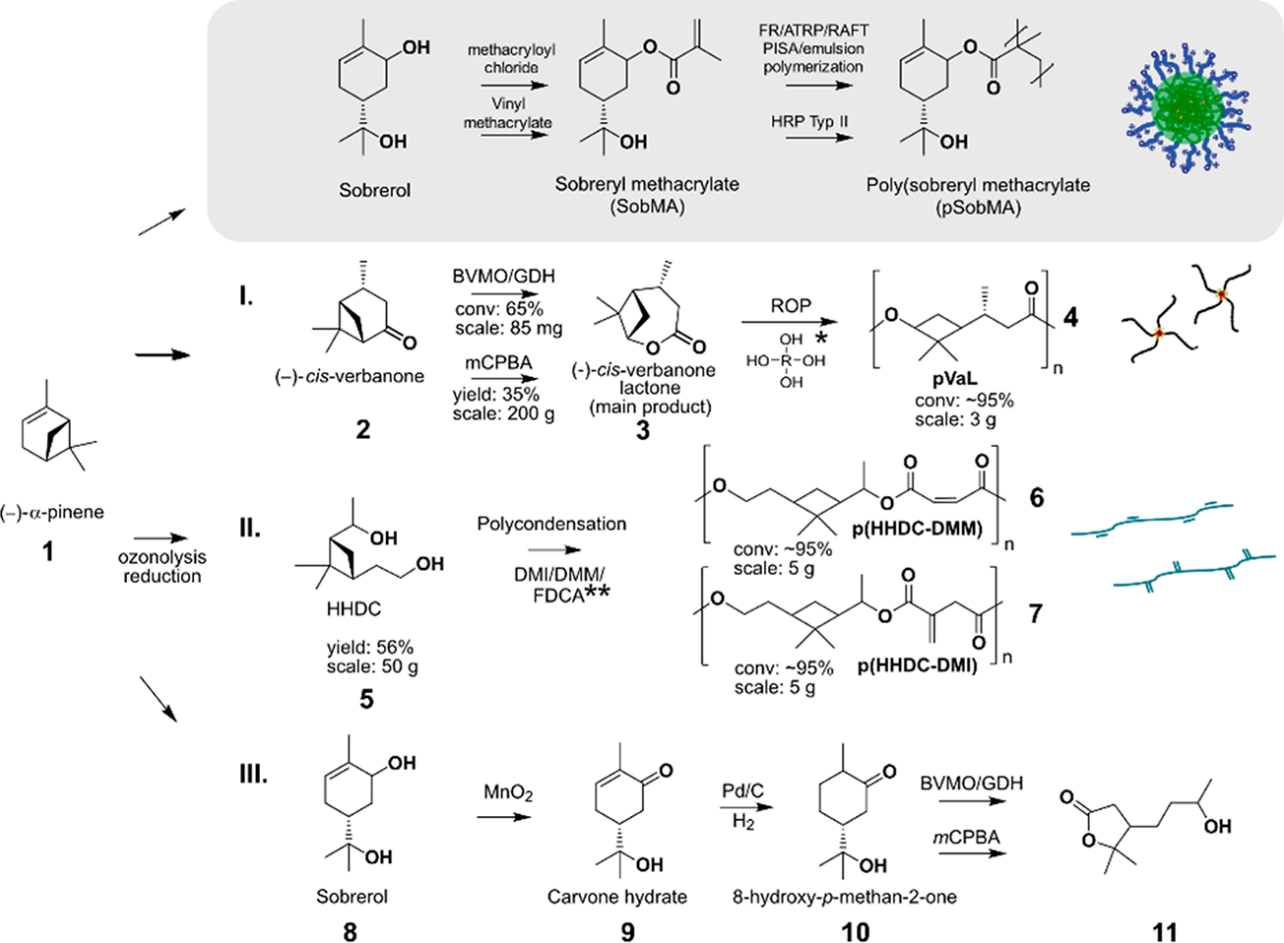
Overview of the (*−*)-α-Pinene-Based Oxidative Synthetic Toolbox for Polyester Generation with Scale and Yields Given Conv, conversion. Gray: previously reported pathways. *Schematic representation of the multifunctional initiator Polyol 4640. **Copolymers containing FDCA are shown in Figure S1.

Terpenes constitute one of the most diversified classes of natural products, with a broad variety of structural and functional properties and with potent applications as bioactive compounds, biochemicals and chiral fine chemical synthons, flavors, and fragrances.^21−23^ A hallmark of terpenes is their (multi)cyclic core that could be preserved into the resulting polymer structure following adequate functionalization.^24^ Incorporation of nonplanar, aliphatic ring units to the polymer backbone can result in several interesting features such as increased elasticity and increased shape recovery, while displaying lower melting temperatures and degrees of crystallinity compared with aromatic polymers,^20,25,26^ all of which are important parameters for controlling material properties. Cyclobutyl rings are incorporated in various natural and pharmaceutical products but are rarely found in industrial materials.^27^ Previous research on poly-α-truxillates showed similar thermal, chemical, and photochemical stability of the cyclobutane-containing polymers compared with polyethylene terephthalate (PET) while offering unique semirigid properties.^27^ The 2007 commercialized Tritan is another successful example of the incorporation of substituted cyclobutane rings into the polymer backbone to create a substitute for bisphenol A (BPA)-containing polycarbonates with a competitive property profile.^28^ Herein, we show the feasibility of polyester synthesis by different mechanisms from the monoterpene (−)-α-pinene, due to its double bond and the presence of a cyclobutyl group that render this metabolite prone to undergo different oxidative reactions to produce monomers compatible with polyester generation. In recent years, the potential of using terpenes as building blocks as the foundation for production of various biobased polymers, such as polyesters, polycarbonates, polyamides, and polyurethanes, has been acknowledged.^29−35^ A recent review on current developments and trends was published by Kleij and Della Monica.^36^ Terpenes have the potential to be isolated in larger amounts due to their abundance in nature and their presence in various industrial side streams.^37,38^ One of the major sources of terpenes is turpentine, a pine-tree resin mixture from the paper and pulping industry with a current annual commercial availability of 350 kt.^39^ In this work, we show how the same biomass-derived terpene metabolite (−)-α-pinene can be transformed into platform monomers by an oxidative toolbox (Scheme 1) for the synthesis of polyesters by both polycondensation and ring-opening polymerization. Chiral biobased polyesters and coatings thereof were produced with high selectivity, scalability, and possible compatibility with additional postpolymer functionalization (Scheme 1). It is envisioned that the conceptually simple oxidative toolbox showcased herein can unlock the reactivity of diverse biomass-derived synthons to generate tailor-made polyesters with properties approaching those of industrially implemented fossil-based materials.

## Results and Discussion

Striving toward renewable building blocks, we aimed to generate oxidative pathways for transformation of (−)-α-pinene into monomers compatible with polyester synthesis (Scheme 1). While only a few terpenes and terpenoid monomers are readily suitable for the synthesis of polyesters, oxidative chemistries such as Baeyer–Villiger oxidation, hydroboration–oxidation, ozonolysis, and epoxidations could unlock the dormant reactivity of terpenes for expedient polyester synthesis. In this scope, chemical and biocatalytic methods to infuse suitable functionalization such as cyclic ester (lactones),^40^ diols,^31^ and epoxides^41^ would be pivotal. In particular, enzymes hold great potential to replace harsh chemical conditions, such as the chemical Baeyer–Villiger transformation associated with toxic reagents and low yields, with mild oxidations in aqueous environment using oxygen as the cosubstrate.^42,43^

To show that oxidative chemistries could pave the way toward generation of biosourced polyesters with both flexible aliphatic- and rigid aromatic-type of motifs, we applied chemoenzymatic catalysis and constructed a route toward star-shaped polyesters (pathway I, Scheme 1). In a second pathway (II), 1-(1′-hydroxyethyl)-3-(2″-hydroxyethyl)-2,2-dimethylcyclobutane (HHDC) was obtained from the oxidative cleavage of the double bound of (−)-α-pinene by ozonolysis (Materials and Methods). This diol was polymerized together with the dimethyl ester of unsaturated or aromatic renewable diacids, such as maleic acid (DMM), itaconic acid (DMI), and furan dicarboxylic acid (DMFDCA). Finally, the versatility of the materials synthesized was demonstrated by subsequent cross-linking of inherent functional groups, using either condensation or thiol–ene coating chemistry.

### Pinene-Derived Polyesters and Star-Shaped Materials Obtained from Ring-Opening Polymerization

We previously demonstrated the potential of using a chemoenzymatic approach to synthesize verbanone (**2**)-based polyesters.^24^ However, the low molecular weight (∼3000 Da), poor control, and low yield in key steps (<40% for generation of cyclic ester **3** from ketone **2** by an engineered Baeyer–Villiger monooxygenase variant)^24^ prevented the full potential of these terpene-based polyesters (**4**) from being achieved. We therefore present an improved route that overcomes those limitations and yields in higher molecular weight materials with good control over the molecular properties.

**Figure 1:**
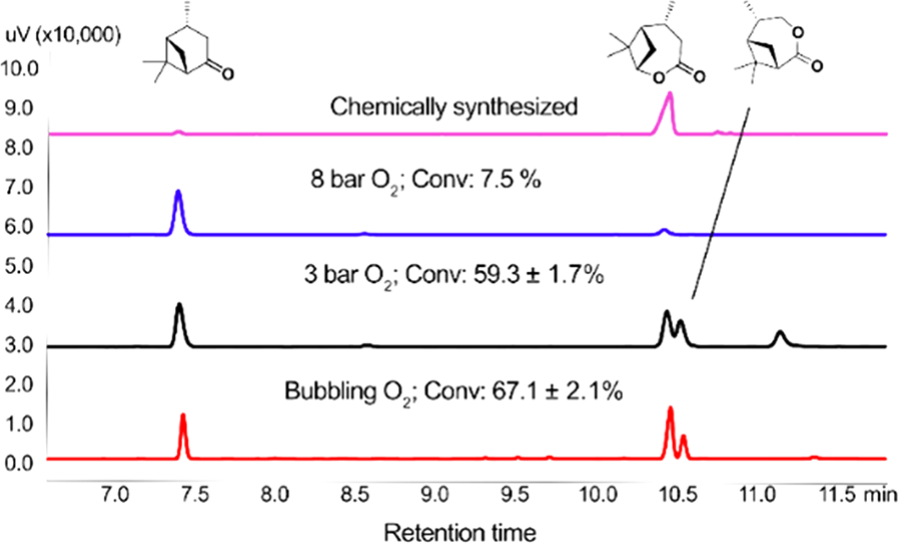
GC-FID of the extracted reaction products of BVMO with verbanone in BR-100 reactor for 24 h (Materials and Methods). All reactions were performed in triplicate to verify the repeatability of the obtained results, in addition to the reaction with 8 bar applied oxygen pressure as duplicates (Table S1). The additional peak at 11.2 min for 3 bar pressure represents an additional side product that we could not identify.

With the aim of achieving efficient biotransformation of (−)-α-pinene-derived ketone **2** into verbanone lactone (VaL) (**3**), we reasoned that oxygen supply is a key factor to achieve efficient enzymatic Baeyer–Villiger oxidation, as recently shown for other members of the oxidoreductase superfamily.^44,45^ Investigations of the ketone **2** conversion ratios under different oxygen conditions were undertaken by performing reactions in a high-pressure stainless-steel reactor varying the oxygen pressure. Utilizing the CHMO_QM_L426A variant^24^ of the Baeyer–Villiger monooxygenase (BVMO) from *Acinetobacter calcoaceticus* (CHMO*Acineto*)^46−48^ previously identified in a screening campaign toward ketone **2**, the results clearly highlight the effect of different oxygen conditions and pressures on the total lactone conversion ratio (Figure 1). Up to 70% of the starting verbanone **2** was observed to be transformed to **3** by continuous bubbling of oxygen at atmospheric pressure, which is significantly higher compared with the previously reported enzymatic conversions by the same variant in a shake-flask (39%).

**Figure 2:**
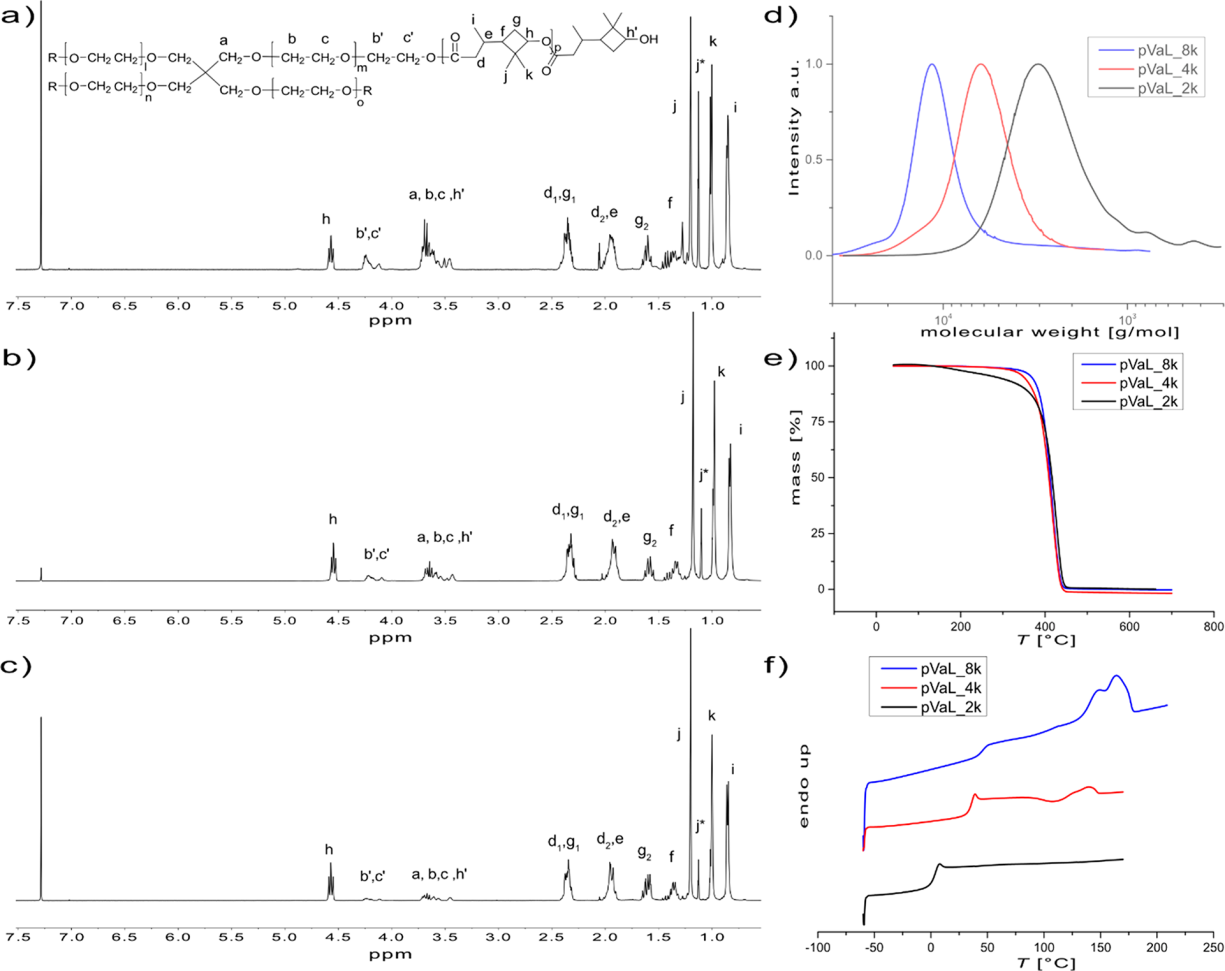
Overview of the material properties of the synthesized pVaLs. ^1^H NMR spectra of (a) pVaL_2k_, (b) pVaL_4k_, and (c) pVaL_8k_. (d) Size-exclusion chromatograms of the respective polymers. (e) Thermogravimetric analysis and (f) differential scanning calorimetry thermograms of the respective polymers.

A further increase of the oxygen pressures to 3 bar resulted in a conversion of around 60%, with appearance of an additional undesired byproduct that we were not able to characterize. However, increasing the oxygen pressure to 8 bar resulted in a dramatic drop in the lactone conversion, which indicates a detrimental effect of the high pressure on the enzyme stability. Additionally, for the production of amounts suitable for material synthesis, chemical BV oxidation was performed using *m*-chloroperbenzoic acid as previously described.^24^ The reaction was herein successfully scaled up to 200 g of verbanone and yielded **3** with ∼35% isolated yield after column purification. One possibility to avoid chlorinated solvents and oxidizing agents was recently presented by Allais et al., who reported a solvent- and catalyst-free Baeyer–Villiger oxidation with just hydrogen peroxide.^49^ The abnormal, least-substituted lactone derived from verbanone was less stable and partly degrades during the base washing steps and on the column, leaving mainly the normal lactone after washup. As an optional path to chromatography that is dependent on large volumes of organic solvents, isolation via distillation yielded in a mixture of normal and abnormal lactones due to similar boiling points. It was possible to separate these two isomers by taking advantage of their inherently different reactivities in selective polymerization of the abnormal lactone from the mixture (a brief discussion on the isolation and polymerization attempts of abnormal lactone is included in the Supporting Information (Figures S2–S5). Polymers of **3** (purified by medium pressure liquid chromatography, MPLC) (pVaLs) were synthesized by ring-opening polymerization reactions in bulk at 70 °C using methanesulfonic acid (MSA) as catalyst (Figure 2 and Table 1). Star-shaped materials could be readily obtained using commercially available ethoxylated pentaerythritol (trade name: Polyol 4640, Figure S6) as a multifunctional initiator. To assess the influence of the chain length on the material properties, three different ring-opening polymerizations were performed, targeting molecular weights (summed over all arms) of around 2000, 4000, and 8000 g/mol. The monomer conversion was determined by ^1^H NMR spectroscopy of the crude reaction mixture using the shift of the epsilon protons from δ = 4.3 to 4.5 ppm, showing monomer conversions above 90% for all polymers. The molecular weights *M*_n_ were measured by size-exclusion chromatography (SEC), and theoretical values were calculated by the ratio between the epsilon protons of the repeating unit (δ = 4.5 ppm) and the terminal methylene protons of the initiator (δ = 3.95–4.25 ppm). The ratio of the polyol methylene protons (δ = 3.95–4.25 ppm) and the combined protons in the region of δ = 3.3–4.3 ppm (32H, polyol protons plus terminal epsilon protons) was further used to calculate the degree of initiation (number of polyol hydroxyl groups that initiated polymer growth), which was calculated to be above 86% (or 3.44 out of 4) for all materials. ^1^H NMR spectra for the three PVaLs are displayed in Figure 2a–c. The molecular weights (*M*_n_) determined by SEC (Figure 2d) were slightly higher for all polymers but still corresponded well with the theoretical *M*_n_ (Table 1). The measured dispersities *Đ* were reasonably narrow although being slightly higher in the case of pVaL_2k_, which can be reasoned to be caused by the statistical larger influence of one single monomer addition in a system with only 2–3 units per polymer arm. Thermal properties of the synthesized materials were studied by thermogravimetric analysis (TGA) and differential scanning calorimetry (DSC) (Figure 2e,f). The thermograms of the synthesized pVaLs show an increased thermal stability with increasing molecular weight and a profile that is in good agreement with the previously published linear pVaL.^24^ The TGA shows that, in the case of pVaL_2k_, mass loss starts already at temperatures just above 100 °C. This low thermal stability of oligomeric polyesters (with molecular weights <3500 g/mol) has been previously reported for materials such as poly(ethylene succinate) and poly(3-hydroxybutyrate),^50,51^ where a low temperature decomposition, accounting for around 15% mass loss, already took place at temperatures above the glass transition temperature of the oligomer.^51^ The degradation temperatures of all polymers (*T*_d_, defined at 5% weight loss) can be found in Table 1. DSC analysis of the materials shows glass transition temperatures of the pVaLs between 2 and 47 °C, with an expected dependency between molecular weight and *T*_g_. The two polymers pVaL_4k_ and pVaL_8k_ further show melting transitions at 139 and 164 °C (Table 1). These thermal properties are in the range of commercially available polylactic acid (PLA), which displays glass transition and melting temperatures in the range of 50–65 and 150–180 °C, respectively.^52,53^

**Table 1:** Summary of the Verbanone-Based Polymers

polymer^a^	reaction time (h)	ratio of VaL (3)/polyol4640/MSA (equiv)	conversion^b^ (%)	initiation efficiency^b^ (%)	*M*_n__,theo_^b^ (g/mol)	*M*_n__,SEC_^c^ (g/mol)	*Đ*^c^	*T*_*g*_^d^ (°C)	*T*_m_^d^ (°C)	*T*_d_^e^ (°C)
pVaL_2k_	0.5	1:0:0.02:0.1	93	86	1920	2080	1.4	2.1		284.7
pVaL_4k_	0.5	1:0:0.02:0.047	95	97	3780	5580	1.2	34.5	139	350.1
pVaL_8k_	0.5	1:0:0.02:0.023	94	100	6780	7300	1.1	46.9	158	368.5

a The subscript numbers refer to the targeted DP.

b Determined by ^1^H NMR.

c Determined by size-exclusion chromatography using chloroform as eluent and polystyrene (PS) standards.

d Determined by differential scanning calorimetry using the second heating to determine the glass transition and melting temperatures.

e Determined by thermogravimetric analysis; *T*_d_ was defined as the temperature corresponding to 5% weight loss. MSA, methanesulfonic acid.

### Unsaturated Terpene-Based Polyester Resins by Polycondensation

In our endeavor to synthesize biobased polyesters, the diol **5** was obtained on a 50 g scale by the oxidative cleavage of the double bond of (−)-α-pinene by ozonolysis (Materials and Methods). The use of ozone for the cleavage of the double bond was previously reported,^54^ still the resulting diol has to the best of our knowledge never been reported in the scope of polyester synthesis. The diol **5** is of particular interest as it allows the synthesis of a variety of biobased polyesters and polyurethanes by reacting it with different diacids or diisocyanides via polyaddition. To present its versatility as a monomer to generate biobased polyesters with different properties, we performed the synthesis of four different polyester compositions, their film formation, and further application as versatile coating materials.

**Figure 3:**
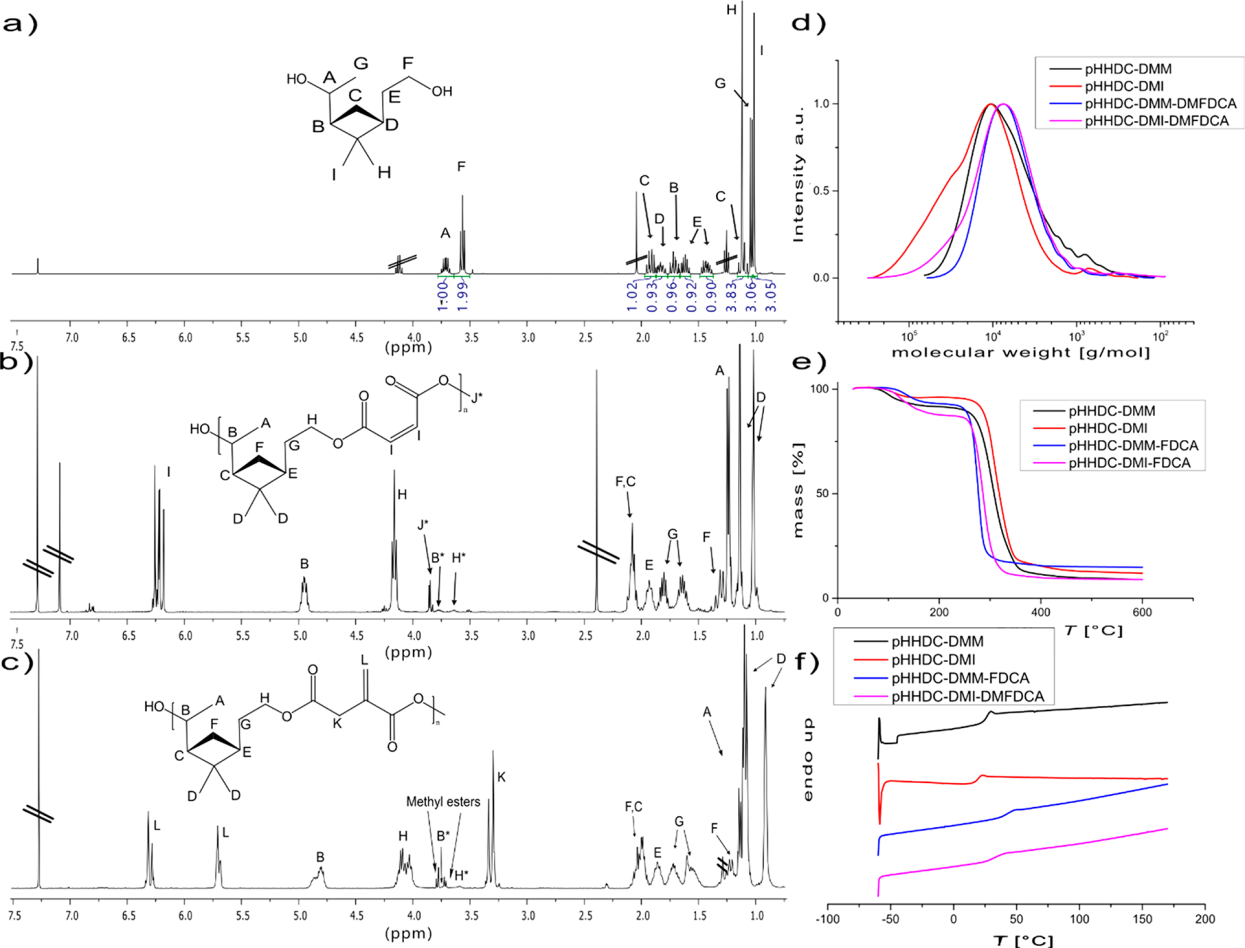
Overview of the material properties of the synthesized HHDC-based polymers. ^1^H spectra of (a) HHDC, (b) pHHDC-DMM, and (c) pHHDC-DMI. (d) SEC of the synthesized polymers. (e) TGA and (f) DSC thermograms of the respective polymers.

Briefly, HHDC **5** was synthesized by oxidative cleavage of the (−)-α-pinene double bond, following the procedure of Tolstikov et al.^54^ The crude product was further purified using MPLC, affording the diol **5** as transparent oil with a yield of 65% (Figure 3a).

**5** was polymerized together with DMM and DMI by condensation polymerization. To further explore the possibility of tailoring different properties of the polymer materials, DMFDCA was added to the polymeric system using a 1:1 ratio of the diesters. In all systems, HHDC was added in a slight excess to the bifunctional esters to prevent an unbalanced reaction due to its higher volatility at the reaction temperature compared to that of the diesters.^55^

The reaction was followed by SEC and stopped once *M*_n_ was above 4 kDa. A representative development of the molecular weight distribution analyzed by SEC is displayed in Figure S7 and shows the typical formation of dimers and trimers in the beginning before larger polymer chains are formed. The polymer was isolated by precipitation with cold methanol and dried under vacuum. Respective ^1^H NMR spectra of the synthesized polymers are displayed in Figure 3b,c and Figure S8.

The molecular weights were further calculated by ^1^H NMR using the vinyl protons (I: δ = 6.25 ppm, L: δ = 6.4–5.7 ppm) together with the protons neighboring the hydroxyl group (B: δ = 4.8 ppm, H: δ = 4.2 ppm) of the repeating unit in relation to the intensity of the signals stemming from respective end groups (δ ∼ 3.5–3.8 ppm). The polymer compositions for the copolymers were assessed by ^1^H NMR and gave [HHDC]/[itaconate]/[FDCA] = 50.6:23.1:26.3 and [HHDC]/[maleate]/[FDCA] = 50.1:23.9:26 (Figure S8). ^1^H NMR also showed an expected conversion of the secondary HHDC-hydroxyl group lower than that of the primary OH for all four polymerizations. The resulting chain ends of the polymers showed hydroxyl functionalities but also methyl esters despite the monomer charging conditions, strengthening the importance of sterics. Exact analysis of end group composition was, however, not possible to determine due to overlapping signals. Molecular weight distribution curves of the four polyesters are shown in Figure 3d.

The two polyesters with itaconate residues show a shoulder toward larger molecular weight. This indication of the occurrence of cross-linking or branching could, however, not be confirmed or quantified by ^1^H NMR.

Thermal characterization of polymers is shown in Figure 3e,f. The TGA thermogram shows a two-step degradation profile for all synthesized HHDC-based polyesters. Similar degradation patterns have previously been published for biobased polyesters containing itaconic acid and FDCA.^56^

Suggested mechanisms of the early polyester decomposition were proposed to include scission of an alkyl oxygen bond. This partial decomposition started around 100 °C for DMI and accounted for up to 15% mass reduction. Full decomposition starts at 240–250 °C for the polyesters containing FDCA, while the itaconate and maleate polyesters have slightly better thermal resistance, decomposing closer to 300 °C. The decomposition temperatures (*T*_d_) of the four polyesters are reported in Table 2.

**Table 2:** Summary of the HHDC-Based Polymers

polymer	monomers (A,B,B*)	reaction time (h)	ratio of *A*/*B*_tot_**/**Ti(OBu)_4_ (equiv)	conversion (A/B/B*)^a^ (%)	conversion of OH′ vs OH″^a^ (%)	product composition^a^ (A/B/B*) (%)	*M*_n__,theo_^a^ (g/mol)	*M*_n__,SEC_^b^ (g/mol)	*Đ*^b^	*T*_g_^c^ (°C)	*T*_d_^d^ (°C)
pHHDC_DMM	HHDC, DMM	7	1.01:1:0.014	97.5:96.9	99.5:95.6	49.8:50.2	6400	4500	2.3	17.0	274.8
pHHDC_DI	HHDC, DI	30	1.02:1:0.014	93.6:97.2	96.1:91.1	50.9:49.1	8400	7700	3.0	13.6	258.5
pHHDC_DMM_FDCA	HHDC, DMM, DMFDCA	7	1.02:(0.5:0.5):0.014	91.9:99.1:99.6	99.7:84	50.6:23.1:26.3	6100	4400	1.9	42.3	153.0
pHHDC_DI_FDCA	HHDC, DI, DMFDCA	16	1.02:(0.5:0.5):0.014	87.1:97.1:95.7	91.2:82.4	50.1:23.9:26.0	6200	4200	2.9	32.5	125.4

a Determined by ^1^H NMR.

b Determined by SEC using chloroform as eluent and PS standards.

c Determined by DSC using the second heating to determine the glass transition temperatures.

d Determined by TGA; *T*_d_ was defined as the temperature corresponding to 5% weight loss.

The DSC thermograms (Figure 3f) show glass transition temperatures below room temperature for pHHDC-DMM and pHHDC-DMI, whereas the polymers containing FDCA display values slightly above room temperature. This increase can be reasoned with the reduced mobility of the FDCA moieties.^57^ DSC shows no exothermic crystallization indicating that all polyesters are fully amorphous.

The possibility of transforming sobrerol **8** into its lactone form, leaving the tertiary alcohol group unreacted during the polymerization and thus ready for postfunctionalization transformations, was further investigated. The lactone was synthesized in a three-step reaction starting from **8** or enzymatically starting from the unsaturated ketone **10**, using the enzymes ene-reductase from *Pseudomonas putida* (XenB) and CHMO*Acineto*. Surprisingly, the produced lactone was not the seven-membered SobL, but the respective five-membered rearrangement product **11** resulting from nucleophilic attack by the tertiary OH (see Supporting Information).

### Coating Preparation

The synthesized polyesters presented in this work bear different functional groups available for postpolymerization modifications. To showcase the possibility of functionalization, cross-linking was performed using condensation and thiol–ene coating chemistry (representation shown in Figure S9).

### pVaL-Based Coatings

To utilize the architecture of the synthesized pVaLs, bearing four terminal hydroxyl groups per molecule, the polymers were cross-linked using hexamethoxymethylmelamine (HMMM), a commonly used cross-linker in thermally curable industrial coating applications. Cross-linker and polyester were mixed with DDBS and dissolved in *n*-butylacetate (xylenes for the highest molecular weight, to facilitate dilution and achieve low viscosity). The coating was applied onto a Teflon substrate and cured at 140 °C to a solid film (Figure S10).

**Figure 4:**
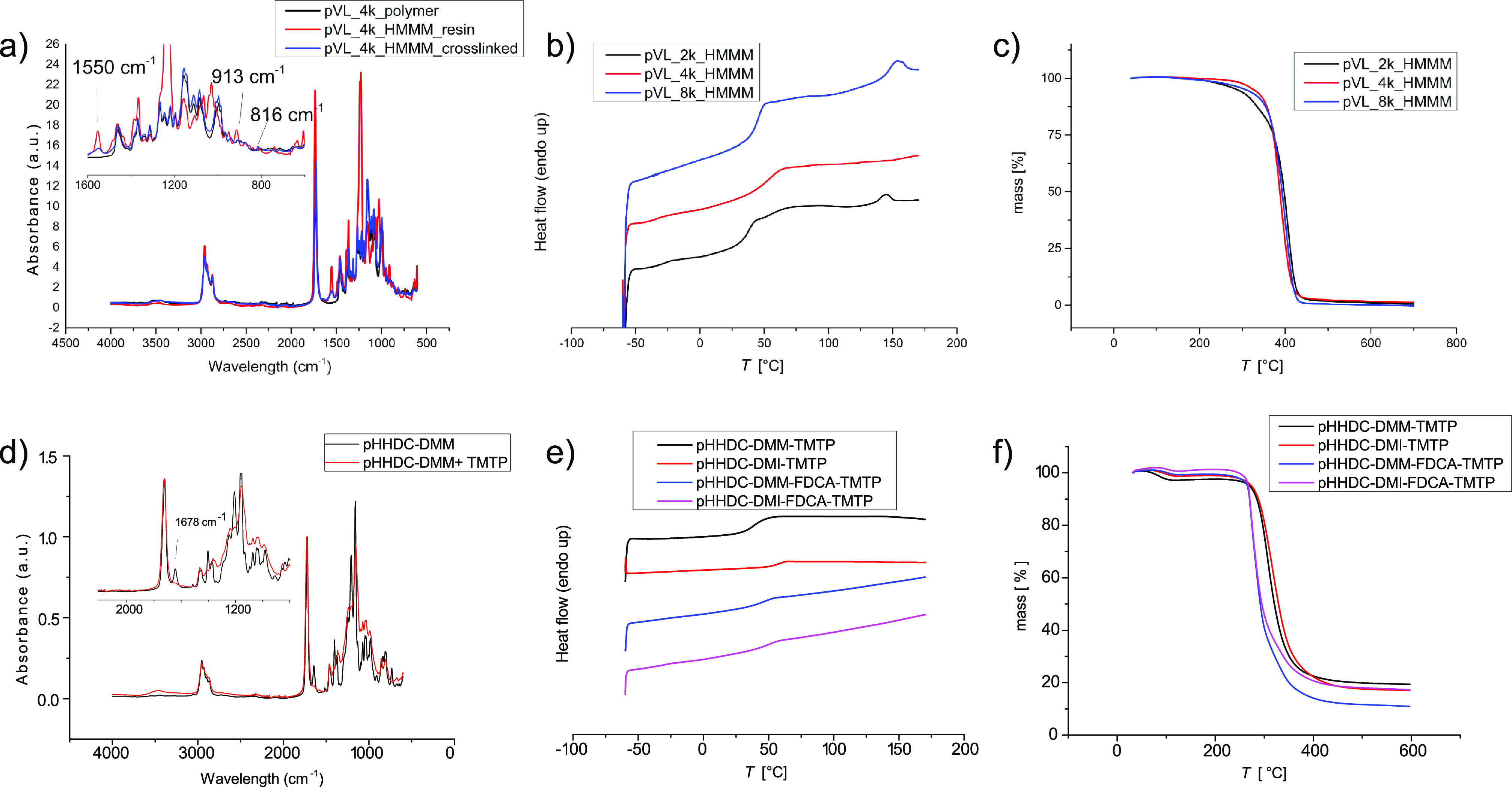
Overview of the material properties of the synthesized HHDC-based polymers. IR spectra of (a) pVaL-based coatings and (d) pHHDC-DMM-based coatings. DSC thermograms of (b) pVaL-based coatings and (e) pHHDC-based coatings. TGA thermograms of (c) pVaL-based coatings and (f) pHHDC-based coatings.

The resulting cross-linked films were analyzed by FTIR spectroscopy (Figure 4a and Figures S11 and S12), which showed clear reductions in the band corresponding to melamine’s methoxymethyl group deformations, compared with native polymer and the resin (913 cm^–1^, normalized to triazine ring deformation at 816 cm^–1^). Additional confirmation of the successful cross-linking is the reduction of the band corresponding to the O–H stretch (∼3390 cm^–1^) as well as the characteristic symmetric band at 1550 cm^–1^, which has been assigned to the quadrant stretching of the triazine ring and contraction of C–N attached to the ring shown to be sensitive toward substitution of the methylol groups.^58,59^

To assess the application of the coatings, thermal analysis was performed on all cross-linked films and compared to the native polymer (Table 3). DSC analysis of the coating (Figure 4b) further confirmed successful cross-linking as both, pVaL_2k_ and pVaL_4k_ exhibited significantly increased *T*_g_ values of 35 and 55 °C, respectively. In case of the films based on pVaL_8k_, no significant influence on the *T*_g_ could be observed. The decrease of crystallinity observed in pVaL_4k_ and pVaL_8k_ is due to the introduced cross-links, limiting the degree of chain movement and preventing crystallization. Further, the decrease of crystalline units also leads to a larger proportion of the material being amorphous, which can be clearly observed in the case of pVaL_8k_.

**Table 3:** Summary of the Coatings

material	M_n_^a^ (g/mol)	cross-linker	method	dry content (wt %)	solvent	*T*_g_^b^ (°C)	*T*_d_^c^ (°C)	CA^d^ (deg)	Cobb120^e^ (g/m^2^)
pVaL_2k_	1920	HMMM	thermal curing	60	*n*-butyl acetate	35.1	287.8	86.1	13.7
pVaL_4k_	3780	HMMM	thermal curing	50	*n*-butyl acetate	55.6	326.9	80.4	4.1
pVaL_8k_	6780	HMMM	thermal curing	50	xylenes	44.8	308	84.0	8.1
pHHDC_DMM	6400	TMTP	UV-curing	40	*n*-butyl acetate	62.0	269.8	72.1	2.7
pHHDC_DMI	8400	TMTP	UV-curing	40	*n*-butyl acetate	57.7	274.1	82.6	1.1
pHHDC_DMM_FDCA	6100	TMTP	UV-curing	40	*n*-butyl acetate	47.1	265.4	61.7	2.1
pHHDC_DMI_FDCA	6200	TMTP	UV-curing	40	*n*-butyl acetate	61.5	267.2	91.8	1.5

a Determined by ^1^H NMR (compare Tables 1 and 2).

b Determined by DSC using the second heating to determine the glass transition temperatures.

c Determined by TGA; *T*_d_ was defined as the temperature corresponding to 5% weight loss.

d Values correspond to the average of five measurements.

e Water absorptiveness is given in g/m^2^; the Cobb tests were made with inspiration from the official ISO-535 standard but with a decreased test diameter of 3.3 cm.

Thermogravimetric analysis showed that, for all films, pyrolysis occurred at lower temperatures compared with the unreacted polymer (Figure 4c). This is primarily due to two reasons, the degradation of unreacted HMMM end groups (140–240 °C) and the degradation of the triazene units (pyrolysis around 400 °C). This decomposition behavior has previously been studied on polyester–melamine coatings, with melamine loadings from 5 to 50%, and this corresponds well to our observations.^60,61^

### HHDC-Based Coatings

All HHDC-based polyesters bear unsaturations in each maleate and itaconate unit. These unsaturations enable postpolymerization modifications, for example, reacting with aliphatic or aromatic thiols to decrease polarity or cross-linking to form gels and coatings.

To assess the possibility of utilizing the unsaturations in the maleate and itaconate units for cross-linking purposes, the polyesters were mixed with trimethylolpropane tris(3-mercaptopropionate) (TMTP, a trifunctional thiol) and a radical initiator (Irgacure 651). The mixture was applied on a glass or Teflon substrate and cured under UV irradiation (Figure S13). The resulting cross-linked films were analyzed by FTIR spectroscopy which showed reductions in the “ene” band, compared with the uncured mixture (CHvC: 1678 cm^–1^, normalized to the carbonyl peak at 1727 cm^–1^; Figure 4d and Figures S14–S16). The successful cross-linking was further corroborated by the fact that the films were insoluble in chloroform and EtOAc, two solvents that readily solubilize the uncured polyesters. The four HHDC-based polyester coatings, where characterized by TGA and DSC analysis to determine thermal properties (Table 3). DSC-derived *T*_g_ values are higher for all four materials compared with the uncured polyesters due to the introduced cross-linkages, hindering coordinated chain movement (Figure 4e). Since the two coatings containing FDCA have only half the amount of unsaturation, the increase in *T*_g_ is less dominant in the respective materials. The *T*_d_-5% obtained from TGA are quite similar for all four materials being around 270 °C (Figure 4f). Curing seems to cause only a slight change in thermal stability compared with the uncured polymers. However, there are little indications of premature decomposition as for the uncured polyesters. The cross-linked network seems to stabilize the weaker components and create a slightly more heat-resistant system.

To test the materials as protective coatings for paper applications, thin ∼10 μm films were coated on industrial used board (ICG280; 280g/m^2^, Iggesund Paper Board) and cured. Results of the Cobb test are all average absorptiveness (*A*) based on three measurements of each sample (Table 3 and Figures S17–S20). To evaluate the obtained values, noncoated board samples were measured (nontreated, UV-treated, and heat-treated). The UV-irradiated board had the highest average *A* value of 51 g/m^2^, whereas nontreated board had 37 g/m^2^. A significant decrease of water absorptiveness is experienced with all coating resins. The two coating formulations including terpene itaconate copolymer even displayed the lowest absorptiveness with 2 g/m^2^. For comparison, disposable food-service items coated with biobased polymers should have a Cobb value lower than 25 g/m^2^.^62^

Contact angle measurement are shown in Figures S21–S23. The contact angle of untreated paper board was 72°. The contact angles of the two references after UV and heat treatment were 52 and 81°, respectively. It shows that while UV irradiation has a negative impact on the paper material inherent properties, heat treating the paper results in increased hydrophobicity.

The pVaL-coated paper showed increased contact angles for all coatings. For the HHDC-based coating, however, different effects could be observed. While the two coatings based on pHHDC-DMM and pHHDC-DMI-FDCA showed decreased wettability of the surface, pHHDC-DMI and pHHDC-DMM-FDCA showed no, or rather the opposite, effect resulting in no improvement compared with untreated paper. However, comparing the contact angle measurements with the results from the Cobb test, two things should be pointed out. First, imperfections in the surface structure of the coating might lead to decreased contact angles. Second, even if lower contact angles point toward increased wettability of the surface, the coating still acts as a protective coating barrier against water absorption. The effect of the surface imperfections on the contact angle was not further investigated.

## Conclusion

Due to resource depletion and climate change associated with human activities, a systemic change of chemical industrial manufacturing is urgently needed,^63^ as this sector currently processes over a billion ton of petroleum-based products annually.^64^ Polymeric materials are essential for our daily life with applications ranging from medicine,^65−67^ electronics,^68−70^ automotive, construction, packaging and preservation of food and beverages.^71−73^ Generation of biobased materials would thus be a key driver for sustainability, a development^63^ which would benefit from the production of a selected panel of biobased monomers fitted to the starting metabolite and that could be combined in different ways to approach properties of fossil-based industrialized materials. Herein, we show how simple oxidative transformations can be used to transform the same terpene metabolite into versatile monomers compatible with different polymerization mechanisms to yield polyesters with different properties.

VaL was synthesized via chemical Baeyer–Villiger oxidation and via enzymatic transformation of verbanone, using an engineered variant of the enzyme CHMO*Acineto*QM previously reported.^24^ Reaction optimization for enhanced oxygen supply showed that the enzymatic transformation efficiency can be increased up to 70% conversion, outperforming the traditional chemical approach. The subsequent polymerization of VaL resulted in star-shaped polyesters with high thermal stability. DSC analysis of the polyesters further showed glass transition and melting temperatures slightly below those of PLA,^52^ with *T*_g_ of 47 °C and *T*_m_ of 158 °C in the case of pVaL_8k_.

Additionally, polyesters were synthesized via polycondensation of the diol HHDC, which was readily obtained from the oxidative cleavage of the double bond in (−)-α-pinene. Unsaturated biobased diesters such as DMM and DMI were used as comonomers to successfully synthesize unsaturated polyester resins. Subsequent cross-linking of the synthesized polyesters by UV irradiation yielded polyester networks with tunable properties.

As an option for extensive transformations to replicate a small set of petroleum-based structures, the presence of multiple functions on a single molecule can allow multiple paths for its valorization by different and fit-for-purpose reaction mechanisms. Simple oxidative transformations can unlock the reactivity of diverse biomass-derived synthons to generate tailor-made polyesters with properties approaching those of industrially implemented materials.

## Materials and Methods

### Materials

All chemicals were obtained from Sigma-Aldrich unless otherwise noted.

#### Recombinant Expression and Crude Extraction of BVMO Protein

The *Acinetobacter calcoaceticus* Baeyer–Villiger monooxygenase variant CHMO_QM_L426A was expressed as described previously.^24^ In brief, a single colony of the *Escherichia coli* BL21(DE3) harboring the CHMO_QM_L426A gene equipped with an N-terminal Histag in a pET28a+ plasmid was pre-cultured overnight in 2× YT media with 40 μg mL^–1^ of kanamycin. The preculture liquid medium was then transferred into a 200 mL fresh 2× YT liquid media in a 1 L baffled flask to a final OD600 of 0.1 and shaken at 37 °C, 200 rpm until the OD600 reached 0.6. The protein expression was induced with 0.05 mM isopropyl β-d-1-thiogalactopyranoside (IPTG) and continually incubated for another 20 h at 180 rpm, 25 °C. Cells were harvested by centrifugation at 4000 rpm, at 4 °C for 20 min, and CHMO QM_L426A cell-free extract (CFE) was prepared with B-PER complete bacterial protein extraction reagent (Thermo Fisher Scientific) following the manufacturer’s instructions. The expression of CHMO was confirmed with sodium dodecyl sulfate-polyacrylamide gel electrophoresis with a gradient 4–15% (Mini-Protean TGX Stain-Free, BioRad, Sweden). The SeeBlue Plus2 (Thermo Fisher Scientific, USA) prestained protein standard was used as a marker. Protein concentration was analyzed by Bradford using the Bio-Rad protein assay (Bio-Rad, Sweden) following the manufacturer’s instruction. Purified CHMO_QM_L426A was prepared by loading the CFE though a His MultiTrap FF column connected to an ÄKTA start protein purification system (GE Healthcare, Sweden). Sodium phosphate buffer (10 mM, pH 7.4) with 500 mM NaCl and 500 mM imidazole was used as the elution solution.

#### BVMO Biocatalytic Reaction

The verbanone conversion reaction was performed using 34 mg of the freshly prepared CHMO_QM_L426A CFE (or optionally, purified protein) with 2 mM verbanone as starting substrate in 50 mM Tris-HCl buffer pH 8.5. Other coenzymes and cofactors include flavin adenine dinucleotide disodium salt hydrate (62 uM), catalase (23120 units), NADPH (0.2 mM), 1.2 mL of glucose dehydrogenase dehydrogenase (240 units), glucose (0.2 mM) prepared in the same buffer and added to BVMO CFE with a total reaction volume of 40 mL, this mixed enzyme solution was kept on ice until use. The reaction was started by adding verbanone to the prepared enzyme solution, and it was immediately poured into the preheated reactor. The reaction was conducted in a Br-100 high pressure reactor (Berghof Products and Instruments GmbH, stainless steel with 100 mL PTFE insert) for 24 h at 30 °C using different oxygen settings.

#### Chemical Synthesis of VaL

The chemical synthesis of VaL was performed in a two-step reaction as described previously.^24^ In the first step verbenone was dissolved in CH_2_Cl_2_ (0.5 M) followed by the addition of 10% (w/w) of Pd/C. The round-bottomed flask was sealed and stirred under H_2_ (≈3 bar) for 4 h. Afterward, the solution was filtered through a p5 glass filter to remove the catalyst. The filtrate was concentrated to afford verbanone **2** in quantitative yields.

*m*-CPBA (2 equiv) was dissolved in anhydrous CH_2_Cl_2_, dried over magnesium sulfate, and concentrated in a round-bottomed flask equipped with a magnetic stirrer under an inert atmosphere. Anhydrous CH_2_Cl_2_ (0.5 mL) and **2** (1 equiv) were added to the flask. The reaction mixture was stirred for 18 h under reflux and an inert atmosphere. After 18 h, the reaction was cooled to room temperature and the resulting slurry was filtered. The filtrate was subsequently washed with saturated sodium bisulfite and saturated sodium bicarbonate, dried over magnesium sulfate, and concentrated. The organic phase was purified by means of MPLC and concentrated to afford the normal most substituted lactone **3** as a colorless oil.

#### Polymerization of VaL

Polyol4640 and MSA were used as received from the supplier. All glassware was dried at 150 °C for 24 h and additionally dried with a heating gun at 600 °C under reduced pressure prior to use. **3** (3 g) was charged into a 7 mL flask together with the polyol according to ratios given in Table 1. The mixture was then subjected to three consecutive vacuum argon cycles. The reaction vessel was placed in a preheated oil bath at 70 °C, and MSA was added under argon. After being stirred for 30 min, the polymers were slightly diluted by the addition of CHCl_3_ and precipitated three times into an excess of MeOH (−78 °C). The recovered polymers (**4**) were dried under reduced pressure overnight.

#### Curing of pVaL-Based Polyesters

Coatings cross-linked via transetherification were prepared by dissolving pVaL (0.50 g), 2-dodecylbenzenesulfonic acid (DDBS, 0.01g), and HMMM (0.14 g for pVaL_2k_; 0.07 g for pVaL_4k_ and 0.04 g for pVaL_8k_) in butyl acetate (0.33 g) or xylene in case of pVaL_8k_. The solution was applied to a glass substrate using a 120 μm applicator and dried in a fume hood for 10 min. The coating was cured in an oven at 170 °C for 15 min.

#### Synthesis of HHDC

The synthesis of HHDC **5** was performed as previously published.^54^ (−)-α-Pinene was dissolved in a 1:1 mixture of MeOH and CH_2_Cl_2_, stirred at −76 °C, purged with an O_3_/O_2_ mixture, and stirred until 1 equiv of O_3_ had reacted. The reaction was afterward purged with oxygen, treated with NaBH_4_, and stirred for 4 h at room temperature. Afterward, hydrochloric acid was added until the pH reached 1. The organic layer was separated, and the aqueous layer was washed three times with ethyl acetate. The organic layers were combined, dried over MgSO_4_, and concentrated under reduced pressure. The crude product was purified using column chromatography resulting in **5** as a transparent oil with a yield of 65%.

#### Synthesis of pHHDC-UPs

Monomers (2.5 g of HHDC) were charged into a 25 mL round-bottom dual-necked flask according to ratios given in Table 2. The attached solvent trap was filled with *p*-xylene. The radical inhibitor mequinol (0.5 wt % of total monomer weight) and 2–3 mL of *p*-xylene were then added. The reaction vessel was connected to a Dean–Stark receiver and lowered into an oil bath at 160 °C. The reaction was started with injection of catalyst Ti(OBu)_4_ (7 mol % of total monomer amount) and carried out for 7–30 h at 1 atm (see Table 2). The resulting polymer was precipitated twice in ice-cold methanol.

#### UV-Curing of HHDC-Based UPs

HHDC-based polymers, thiol agent TMTP (1:1 molar ratio of thiol to unsaturated double bonds), UV-curing agent Irgacure 651 (1 wt % of resin weight), and solvent butyl acetate (40 wt % of resin weight) were mixed and then poured onto the substrate. All coatings were applied with box applicators. For free-standing films, a box applicator with 120 μm thickness was used, and for carton substrates, 30 μm was used. Upon being dried in a fume hood for 10 min, the coating was cured by UV irradiation (10 × 5 s, total dose ∼ 30 J cm^–2^).

#### Cobb Test

The Cobb test was made with inspiration from the official ISO-535 standard. Due to the limited width of the applicators present, instead of a metal cylinder with clamping potential and a diameter of 10 cm, a rubber cylinder with an inner diameter of 3.3 cm was used. Instead of clamping, a circular metal weight was used to fixate the cylinder. To determine baseline absorptiveness, three pieces of untreated carton where subjected to the Cobb120 test. Additional sets of cartons were treated with the respective cross-linking conditions to account for possible substrate effects on absorptiveness post curing.

### Instrumentation

GC-MS (Shimadzu, GCMS-QP2010 Ultra) was performed on an Rxi-5 ms column (30 m, 0.25 mm [inner diameter], 0.25 μm [film thickness], RESTEK). The temperature program was set at 70 °C before being increased to 300 °C with a rate of 20 °C/min and finally increased to 350 °C with a rate of 5 °C/min before being held at 350 °C for 10 min. For MPLC, a Biotage Isolera Four system equipped with a UV detector and Biotage KP-Sil SNAP cartridge columns was used. ^1^H (400 MHz) NMR spectra were recorded with a Bruker Avance AM 400 instrument. The signal of the deuterated solvent CDCl_3_ (δ = 7.26 ppm^74^) was used as reference. For SEC, a TOSOH EcoSEC HLC-8320GPC system was used equipped with an EcoSEC RI detector and three PSS PFG 5 μm columns (microguard, 100 Å, and 300 Å). Polystyrene standards were used for calibration and chloroform as eluent; toluene was used as internal standard. DSC was performed using a Mettler Toledo DSC 820 module. Samples (5–10 mg) were prepared in 100 μL aluminum crucibles. The samples were subjected to heating from 30 to 170 °C (or 160 °C), cooled to −60 °C, and then heated again to 170 °C (or 160 °C) at a heating/cooling rate of 10 °C/min under nitrogen flow (50 mL/min). The data obtained from the second heating were used for analyses. For TGA, a Mettler Toledo TGA/DSC1 instrument was used. Samples (5–7 μg) were prepared in 70 μL alumina crucibles and heated from 40 to 700 °C at a heating rate of 10 °C/min under a nitrogen flow (50 mL/min). FTIR was performed on a PerkinElmer spectrum 100 FTIR instrument equipped with a single reflection ATR system and a MIR-TGS detector using a MKII Golden Gate (Graseby Specac Ltd., Kent, England). Spectra were recorded over the range 4000–600 cm^–1^ and based on 16 scans at an average resolution of 4.0 cm^–1^.

## Supporting Information Available

^1^H NMR spectra, FTIR spectra, contact angle measurements, supplementary figures and text (PDF)
