# Supplementary material for: Pinene-Based Oxidative Synthetic Toolbox for Scalable Polyester Synthesis
Source: JACS Au. 2021 Oct 8;1(11):1949–60. doi: 10.1021/jacsau.1c00312 (PMC8620555; doi:10.1021/jacsau.1c00312)
Supplement: Supplementary file 1 — au1c00312_si_001.pdf [file au1c00312_si_001.pdf]

Supporting information

Pinene-based oxidative synthetic toolbox for scalable polyester synthesis

Arne Stamm,[a] Johannes Öhlin,[a] Caroline Mosbech,[a] Peter Olsén,[a] Boyang Guo,[b] Elisabeth Söderberg,[b] Antonino Biundo,[b] Linda Fogelström,[a,c] Shubhankar Bhattacharyya,[d] Uwe T. Bornscheuer,[e] Eva Malmström[a,c] and Per-Olof Syrén.*[a,b,c]

AUTHOR ADDRESS

[a]KTH Royal Institute of Technology, School of Engineering Sciences in Chemistry, Biotechnology and Health, Department of Fibre and Polymer Technology, Division of Coating Technology, Teknikringen 56-58, SE-100 44 Stockholm, Sweden. E-mail: per-olof.syren@biotech.kth.se.
[b]KTH Royal Institute of Technology, School of Engineering Sciences in Chemistry, Biotechnology and Health, Science for Life Laboratory, Tomtebodavägen 23, Box 1031, SE-171 21 Solna, Sweden.
[c]KTH Royal Institute of Technology, School of Engineering Sciences in Chemistry, Biotechnology and Health, Department of Fibre and Polymer Technology, Wallenberg Wood Science Center, Teknikringen 56-58, SE-100 44 Stockholm, Sweden.
[d]RISE Processum AB, Hörneborgsvägen 10, Domsjö-89258 Örnsköldsvik, Sweden.
[e]University of Greifswald, Institute of Biochemistry, Department of Biotechnology and Enzyme Catalysis, Felix-Hausdorff-Strasse 4, 17487 Greifswald , Germany.

Table of Contents

1. **Polycondensation of FDCA-containing polyesters** 2
2. **Enzymatic synthesis of the verbanone-derived lactone** 2
3. **Polymerization of the abnormal verbanone lactone** 2
4. **Polyol4640** 6
5. **HHDC Copolymers** 7
6. **Crosslinking of the pinene-based polyesters.** 8
7. **Verbanone-based coatings** 9
8. **HHDC-based coatings** 11
9. **Cobb-test and contact angle analysis** 13
10. **Synthesis of sobrerol lactone** 18

1. Polycondensation of FDCA-containing polyesters

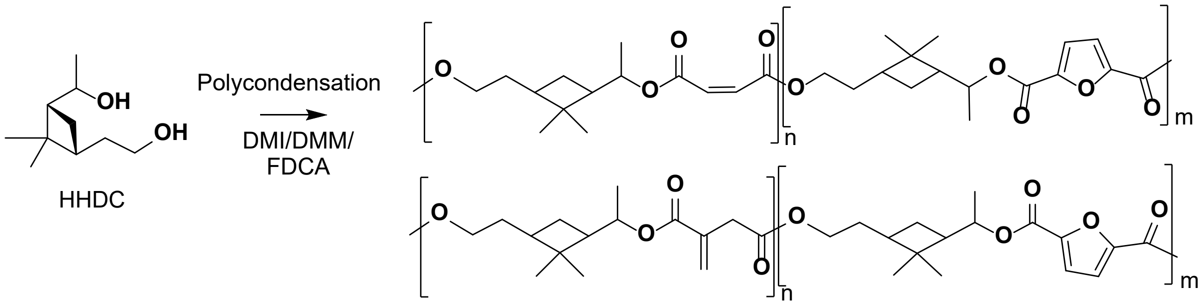

Figure S1. Schematic structure of the FDCA-containing copolymers. The ratio between and FDCA and DMI and DMM was set to 1:1 respectively.

2. Enzymatic synthesis of the verbanone-derived lactone

Table S 1. Conversion in the BVMO-catalyzed oxidation of verbanone **2**. Conversion was determined by GC-FID after 24 h from the extracted crude reaction product**.**

Condition	Reaction	Conversion [%]	Average conv. [%]	std dev. [%]
O_2-Bubbling	CM14	70	67.1	2.1
	CM16	66		
	CM21	66		
3 bar O_2	CM2	57	59.3	1.7
	CM4	60		
	CM12	61		
8 bar O_2	CM5	4	7.5	
	CM6	11		

3. Polymerization of the abnormal verbanone lactone

To obtain a polymer based on the abnormal verbanone lactone, a small amount of ethanol was added to the mixture of the normal and abnormal lactone and the temperature was set to 50 °C to polymerize the more unstable abnormal lactone out of the mixture (Figure S2a: ^{1}H-NMR of the mixture obtained from distillation). The polymerization was conducted for about 6 hours. The polymer could then be precipitated to separate the polymer based on the abnormal lactone from the mostly unreacted normal verbanone lactone. The polymer was then precipitated a second time to remove residual monomers and characterized by ^{1}H-NMR and SEC. The ^{1}H-NMR of the polymer is shown in Figure S2b. To calculate the theoretical molecular weights, *M*n, the ratio between the epsilon protons of the repeating unit (abnormal lactone: d= 4.0 ppm and 3.6 ppm; normal lactone: 4.6) and the ethanol protons (d = 4.15 ppm, CH_2) were used, resulting in an average *M*n of 3100 g/mol. The ^{1}H-NMR further showed partial polymerization of the normal lactone. However, the isolated polymers contained more than 80% of the abnormal lactone, showcasing the potential to separate the two lactones by polymerizing the abnormal lactone out of the distillate. To analyse the end groups of the synthesized material, the polymers were reacted with trifluoroacetic anhydride in DCM. The ^{1}H-NMR of the resulting end-capped polymers is shown in Figure S2c and reveals that the normal lactone was the dominant end group. It should be noted that the molecular weight in those samples occurs higher due to several washing steps that were conducted to remove the triethylamine and pyridine.

The molecular weight distribution was further measured by size exclusion chromatography (Figure S3). The obtained molecular weight Mn of the polymer was around 2.4 kg/mol, which is in good agreement to the molecular weight detected by NMR.

To investigate the thermal properties and the influence of the stereochemistry on the resulting material properties, DSC analysis was conducted on the polymer. The profiles of the cooling and the second heating cycle at different heating rates are shown in Figure S4. The polymer of the abnormal Lactone exhibited semi-crystallinity with a Tm around 150°C and a glass transition temperature around 50°C. The cold crystallization peak in the thermograms with 10 and 20°C/min in the heating curve at around 100°C is a result of the slow crystallization behaviour of the polymer most probably related to its bulky chemical structure. To visualize this effect, films were coated on a glass substrate. The glass substrate was then heated to 170°C for 10 min and rapidly cooled down by placing the glass substrate in the freezer. Afterwards the films were heated to 70°C for 30 min to allow the material to crystallize (Figure S5).

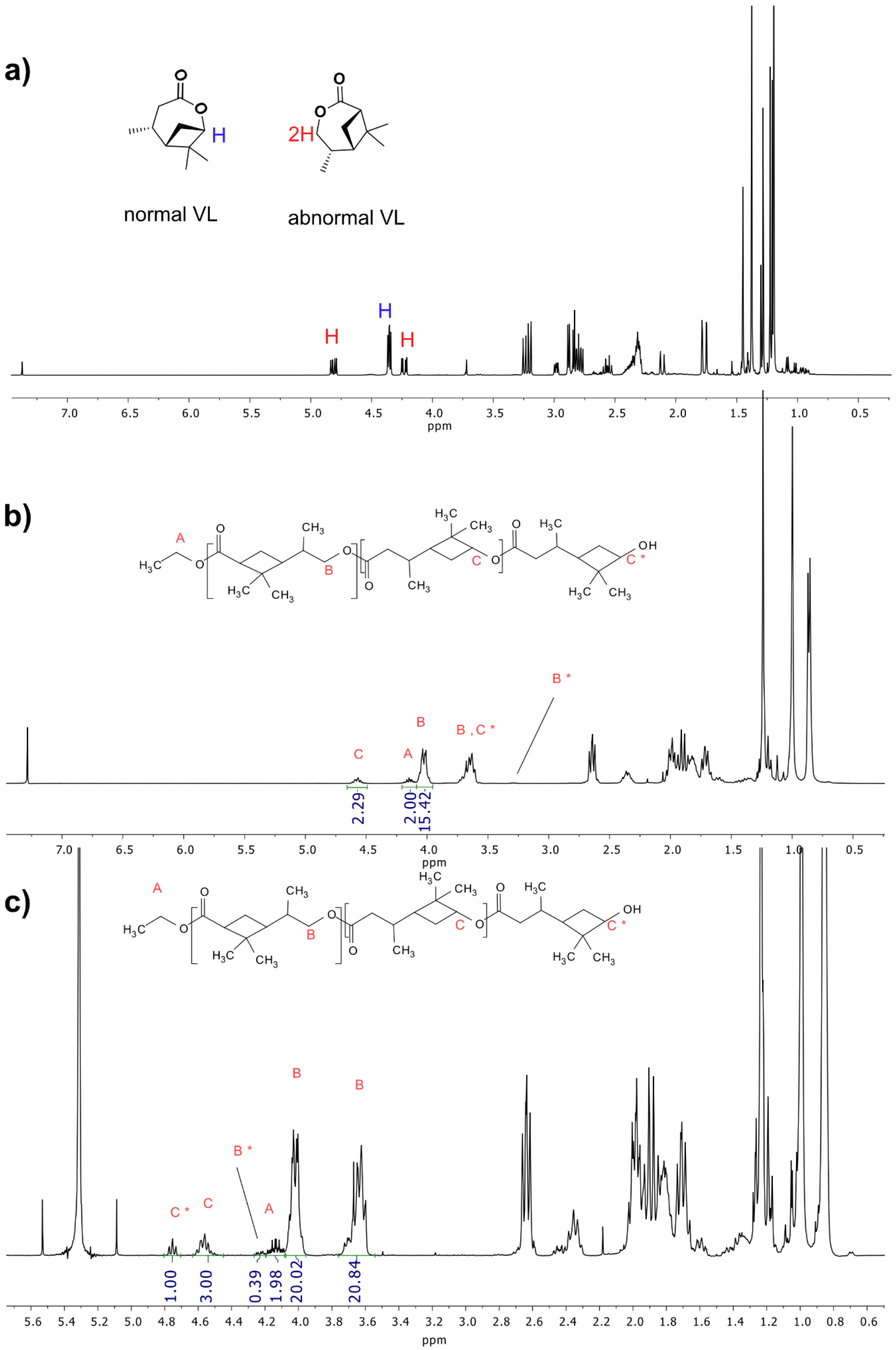

Figure S2. a) ^{1}H-NMR of normal and abnormal lactone obtained from distillation; b): ^{1}H-NMR of the purified polymer obtained from polymerizing the abnormal lactone and c) polymer reacted with trifluoroacetic anhydride to identify end groups.

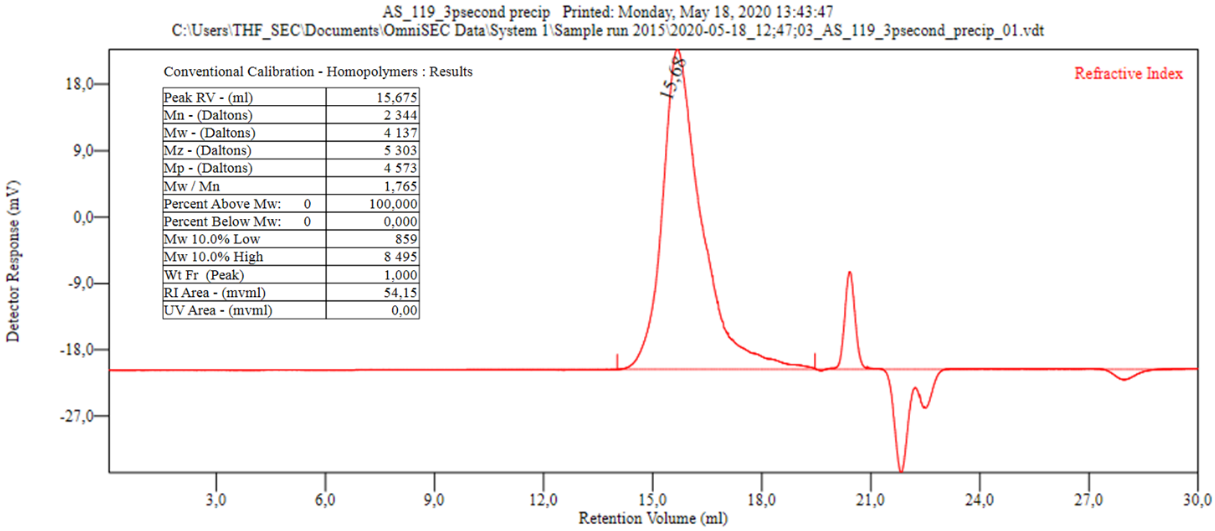

Figure S3. SEC result for the abnormal polymer.

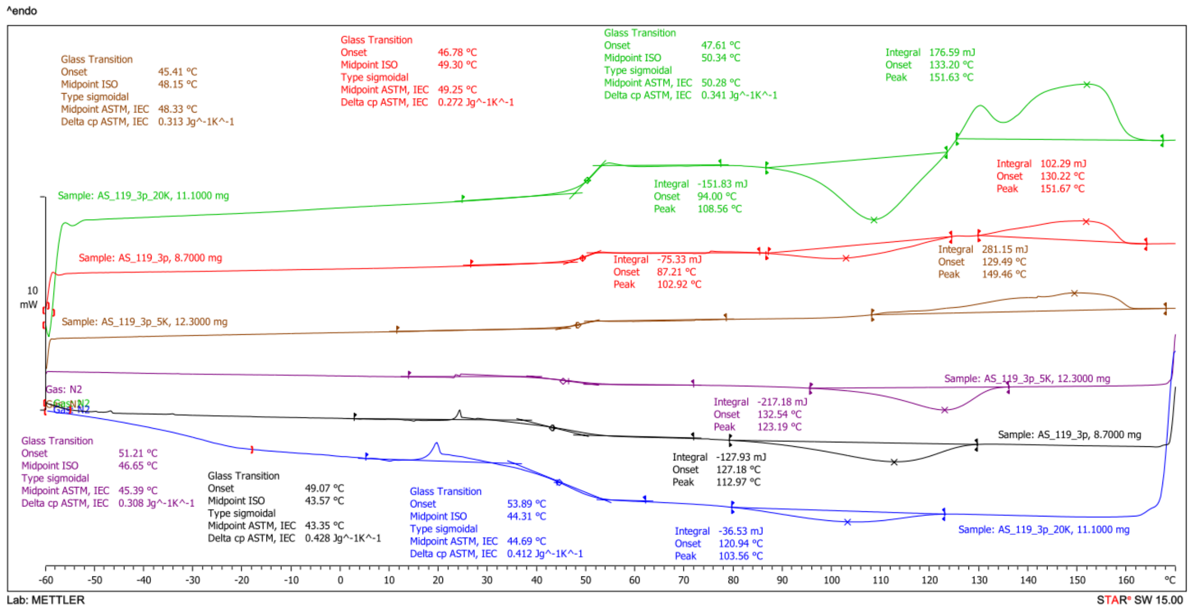

Figure S4. DSC thermograms of the abnormal lactone at different heating rates.

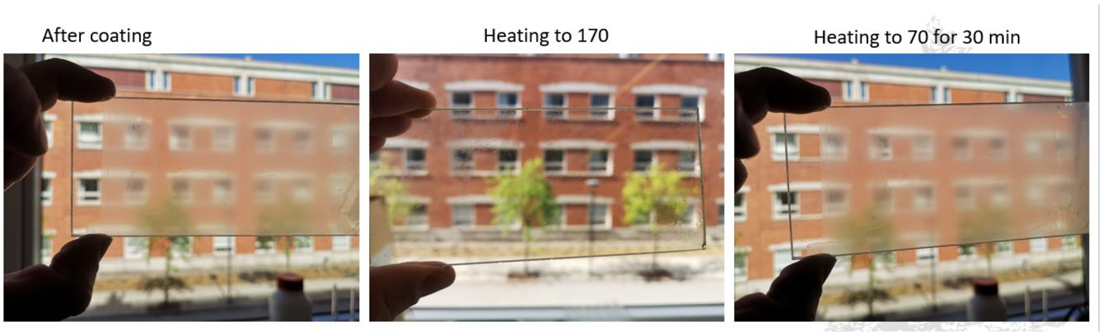

Figure S5. Influence of crystallization of the polymer based on the abnormal lactone on polymer transparency.

4. Polyol4640

To assess the amount of ethoxy groups per molecule, polyol 4640 was reacted with trifluoroacetic anhydride in the presence of pyridine. The reaction was conducted in DCM at room temperature overnight. To stop the reaction, DCM was added, and the solution was washed twice with brine. The downfield shift of terminal methylene protons (signal around 4.5 ppm) was then used to calculate the ratio of terminal/nonterminal protons. Based on the assumption of complete functionalization of all hydroxyls, the amount of ethoxy groups was determined to be **l+m+n+o=5**, which is in good agreement with the nominal molecular weight reported by the manufacturer of around 360 g/mol.

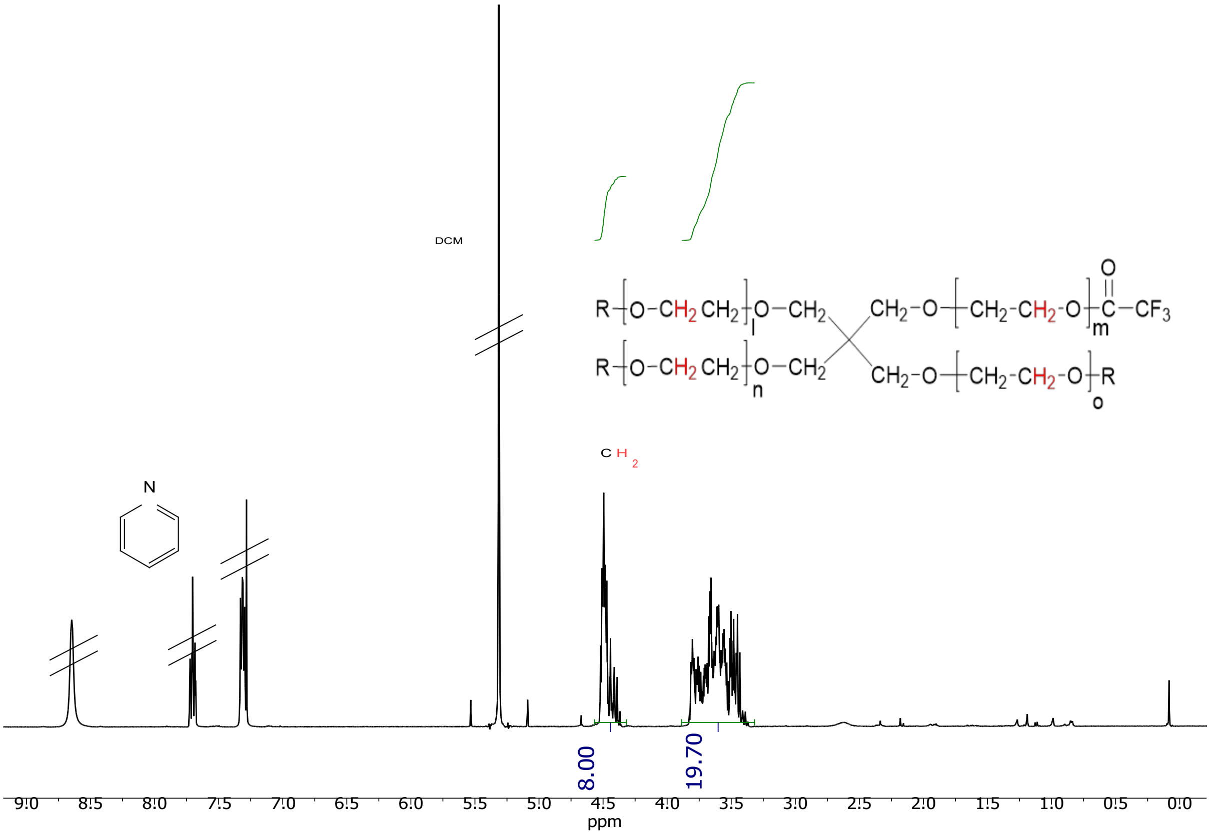

Figure S6. ^{1}H-NMR of the TFA-functionalized polyol4640. Terminal methylene protons are marked in red.

5. HHDC Copolymers

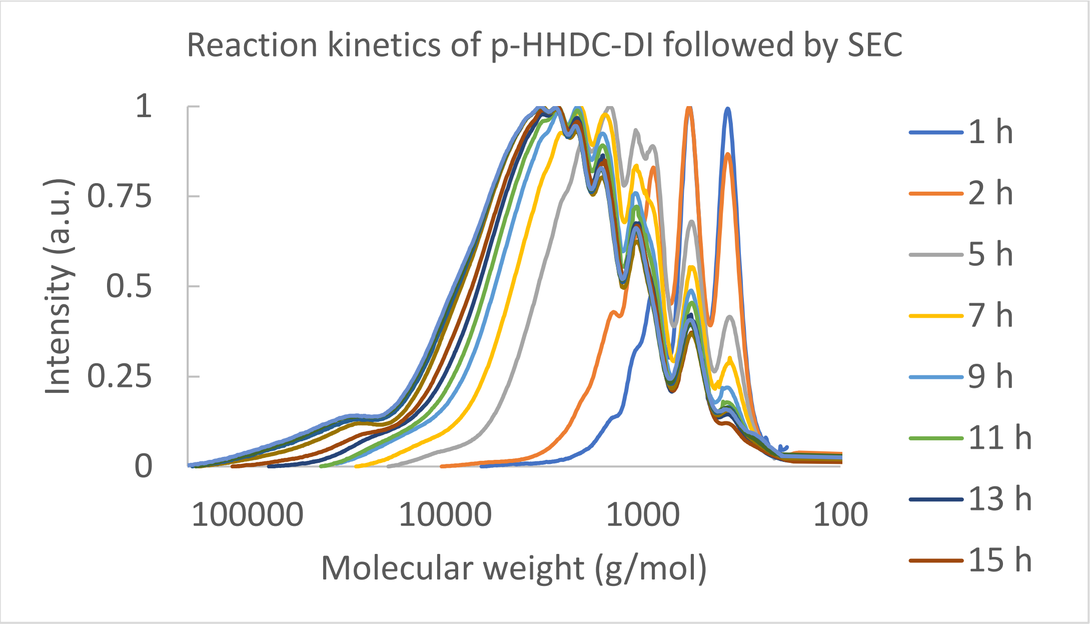

Figure S7. SEC-traces of aliquots taken at different time points.

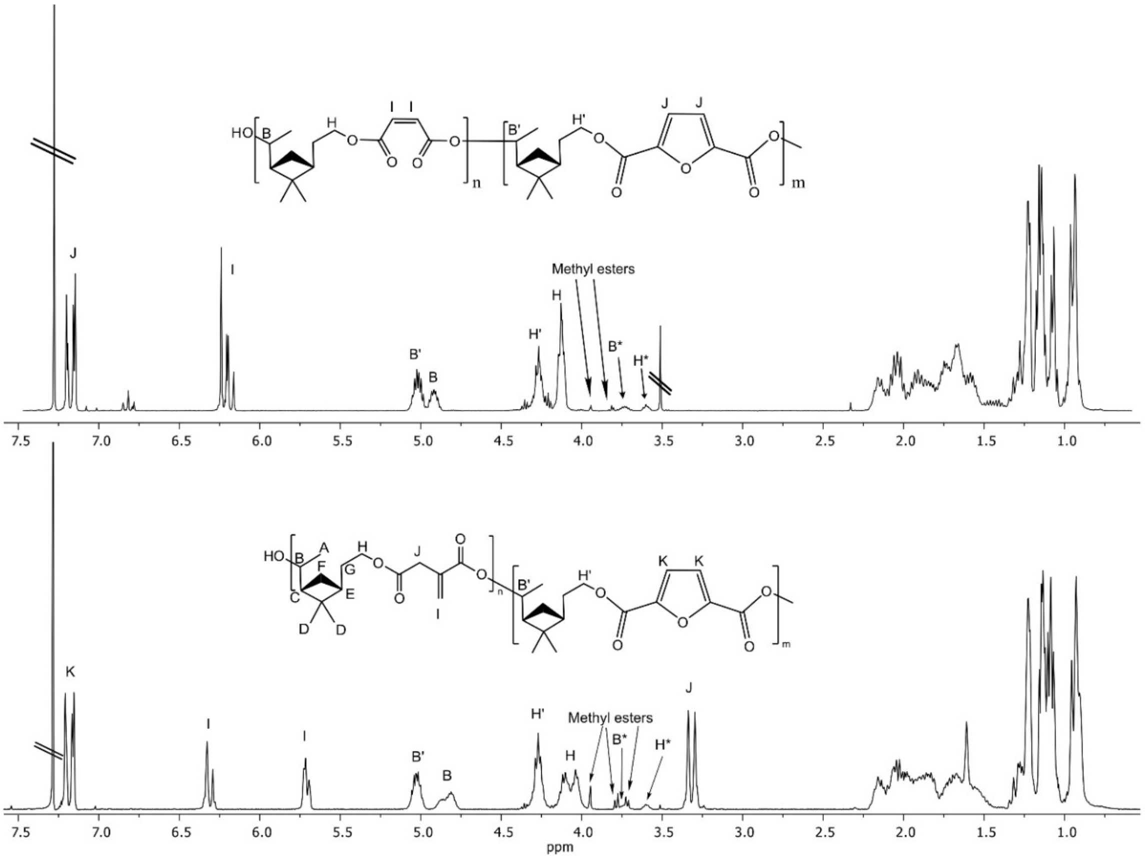

Figure S8. ^{1}H-NMR of the synthesized copolymers including FDCA.

6. **Crosslinking of the pinene-based polyesters.**

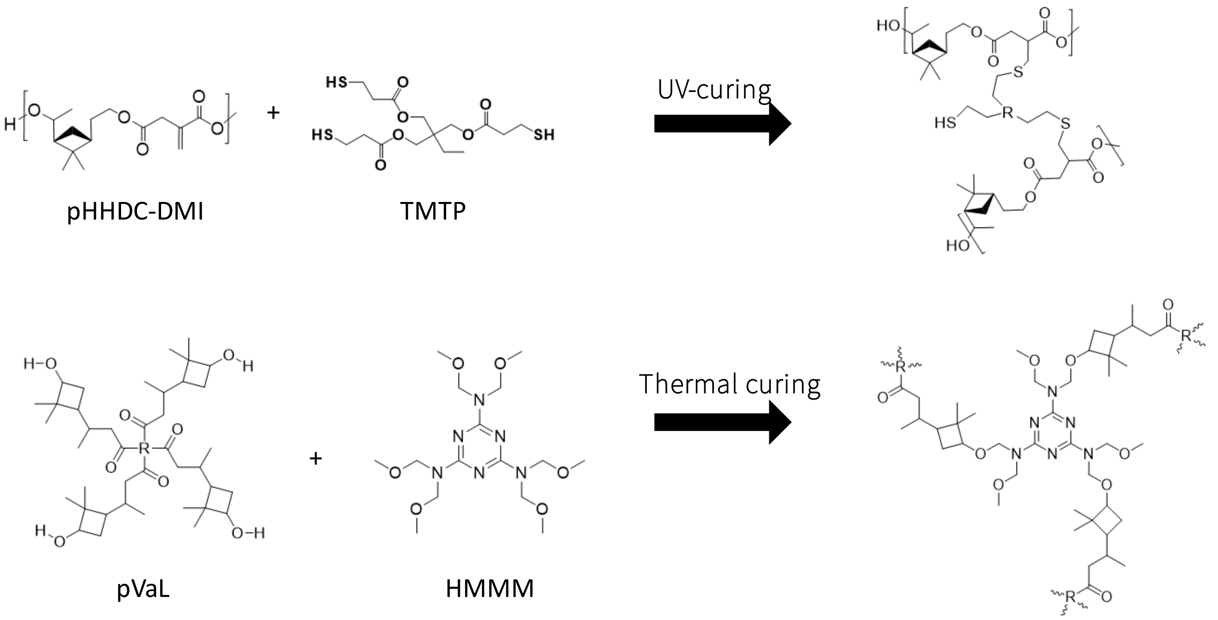

Figure S9. Schematic representation of the post-polymerization functionalization by UV-curing and thermal curing. The structure of pVaL was simplified in order to increase clarity, correct pVaL structure is displayed in Fig 2 of the manuscript.

7. Verbanone-based coatings

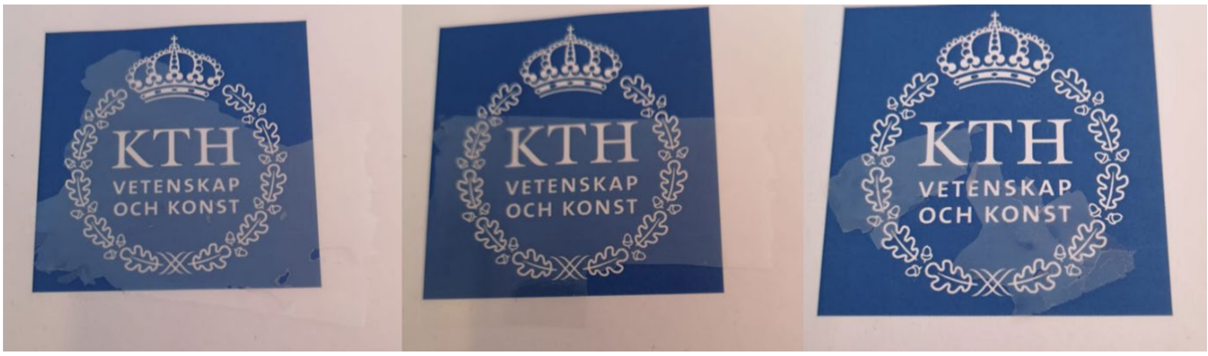

Figure S10. Pictures of the crosslinked pVaL films. From left to right: pVaL_2k, pVaL_4k, pVaL_8k. KTH logo reproduced with permission from KTH.

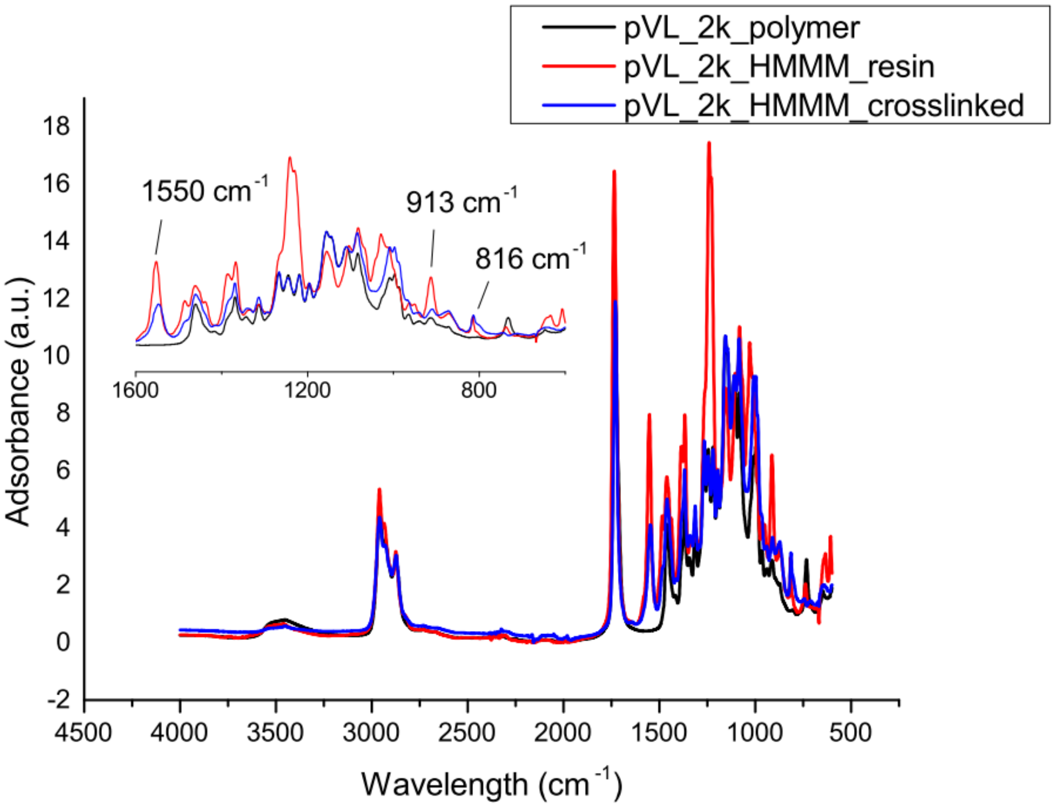

Figure S11. FTIR of pVaL_2k, characteristic melamine peaks are marked. Note that for the names of experiments given in the box, pVL is synonymous to pVaL (scheme 1 in main paper).

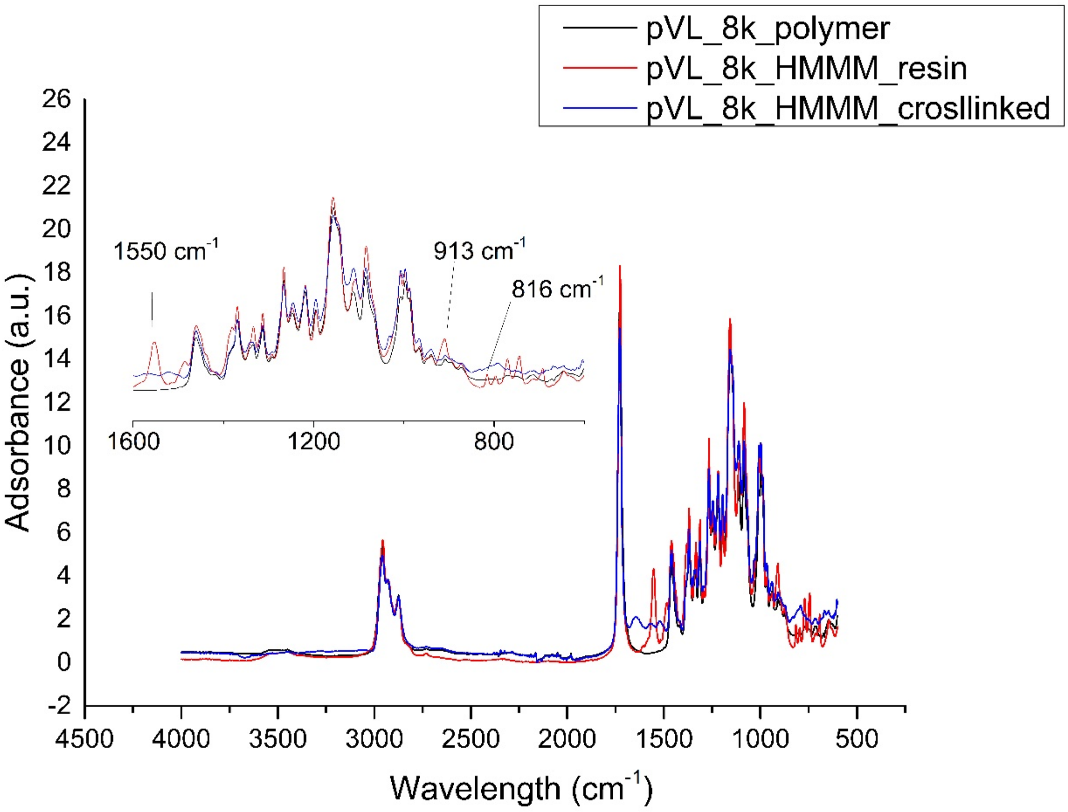

Figure S12. IR of pVaL_8k, characteristic melamine peaks are marked. Note that for the names of experiments given in the box, pVL is synonymous to pVaL (scheme 1 in main paper).

8. HHDC-based coatings

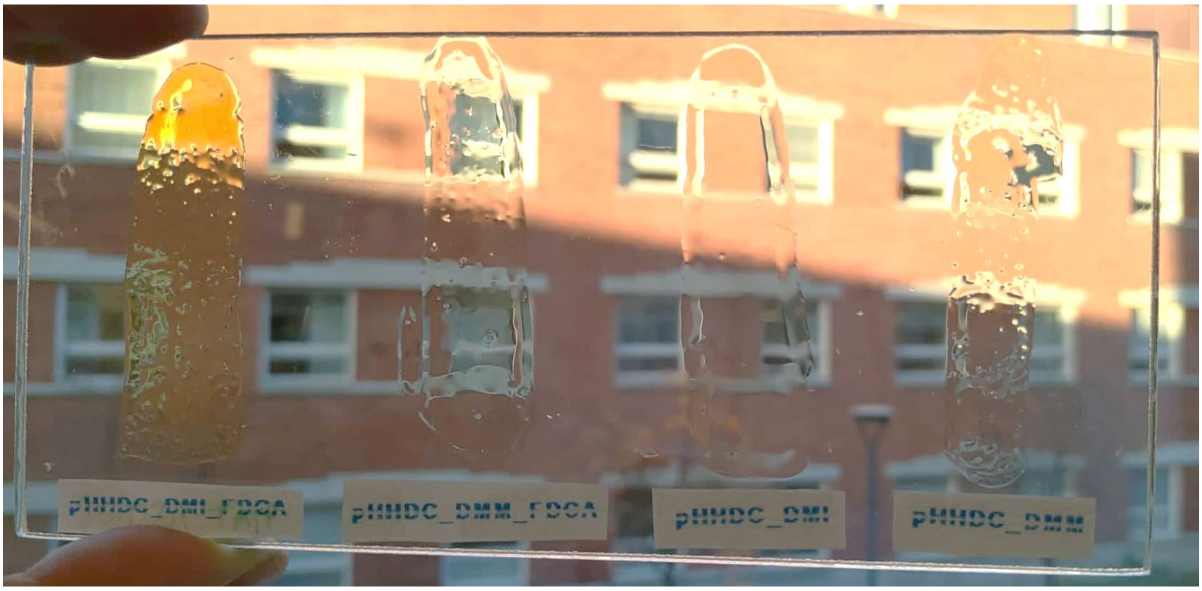

Figure S13. HHDC-based films applied on glass substrate.

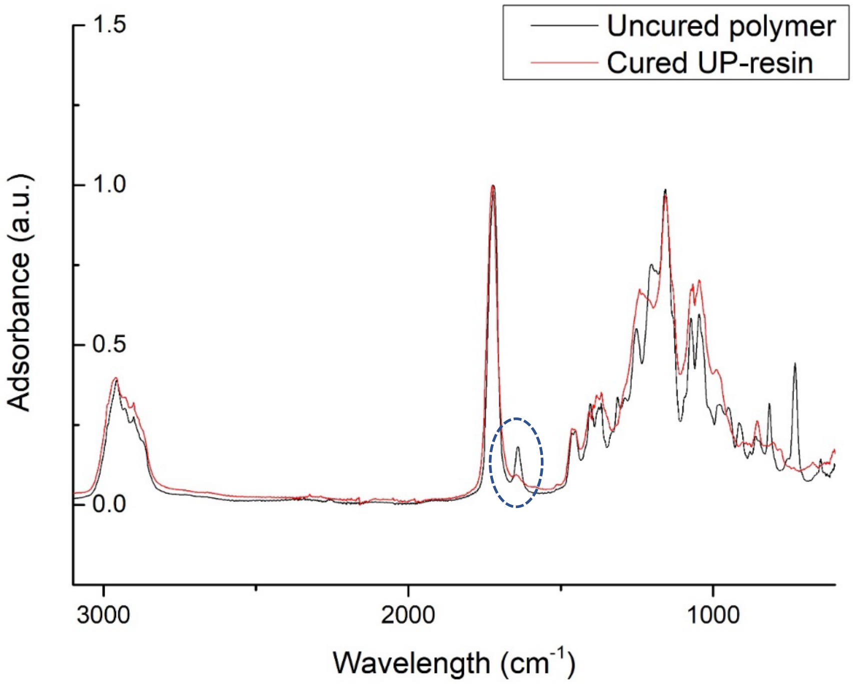

Figure S14. FTIR of pHHDC-DMI, the C=C-signal at ~1640 cm^{-1} is highlighted. The post-cure signal decrease indicates that the majority of double bonds has been consumed in the curing reaction.

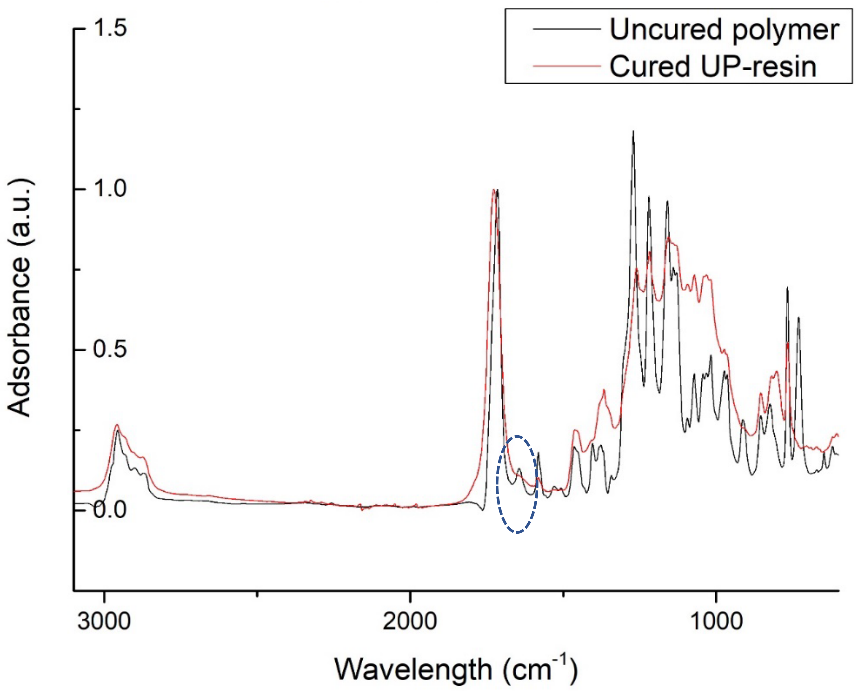

Figure S15. FTIR of pHHDC-DMM-FDCA, the C=C-signal at ~1640 cm^{-1} is highlighted.

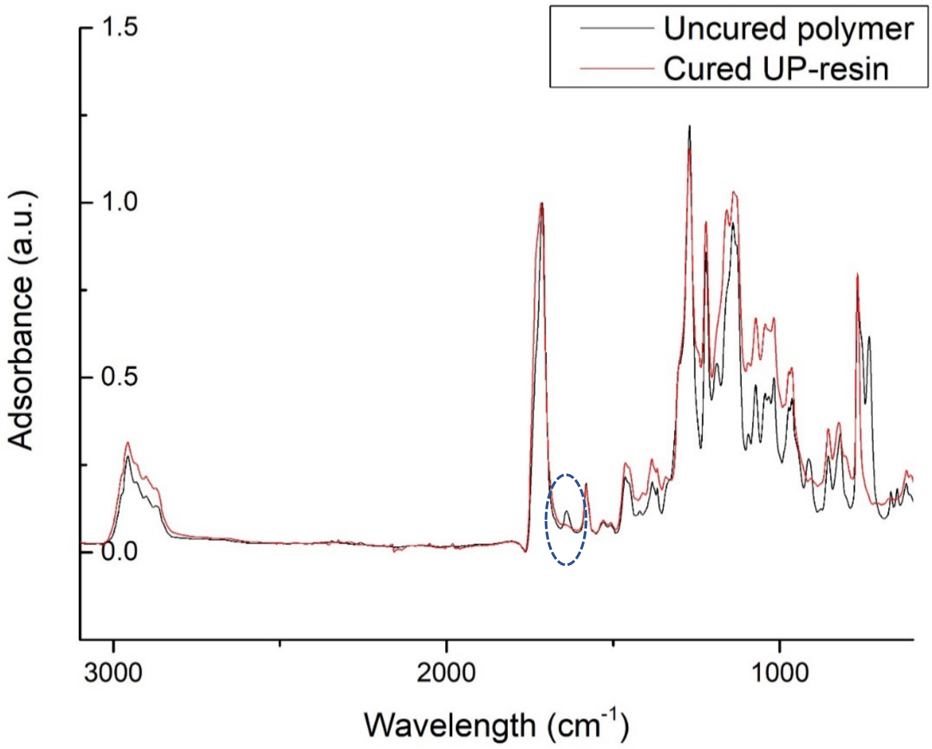

Figure S16. FTIR of pHHDC-DMI-FDCA, the C=C-signal at ~1640 cm^{-1} is highlighted.

9. Cobb-test and contact angle analysis

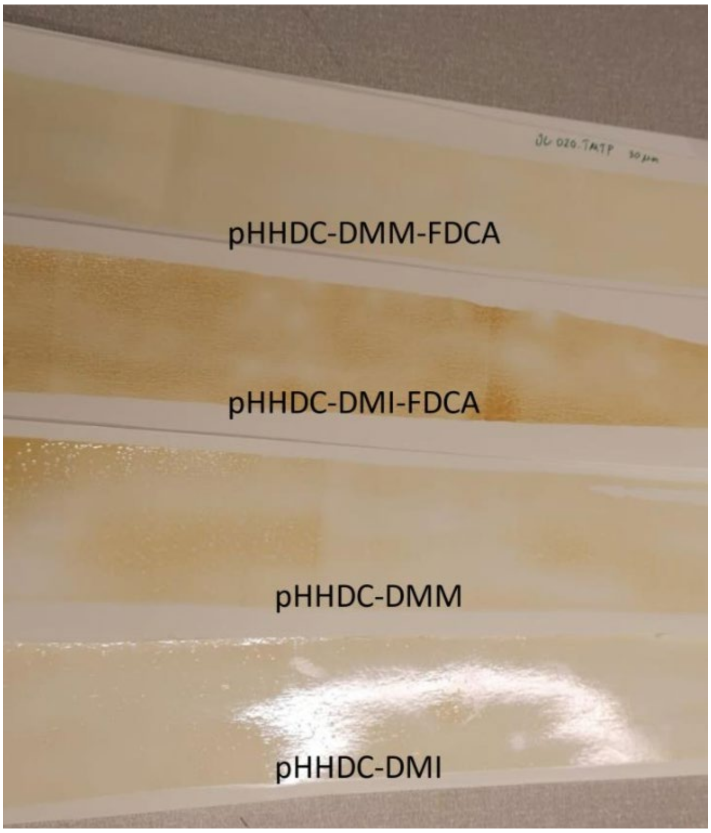

Figure S17. HHDC based coatings on paper.

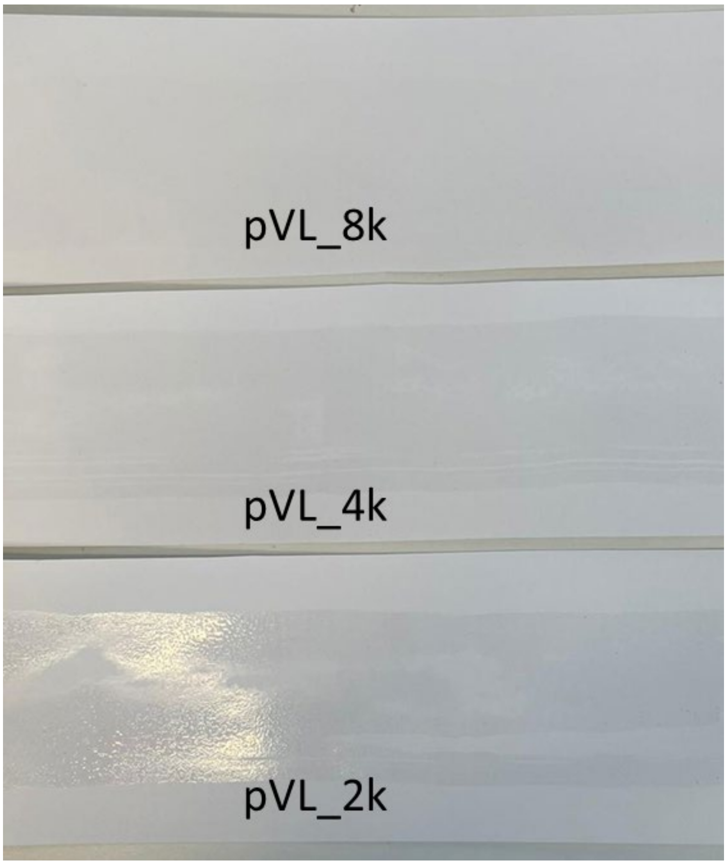

Figure S18. Verbanone-based paper coatings. Note that for the names of experiments shown, pVL is synonymous to pVaL (scheme 1 in main paper).

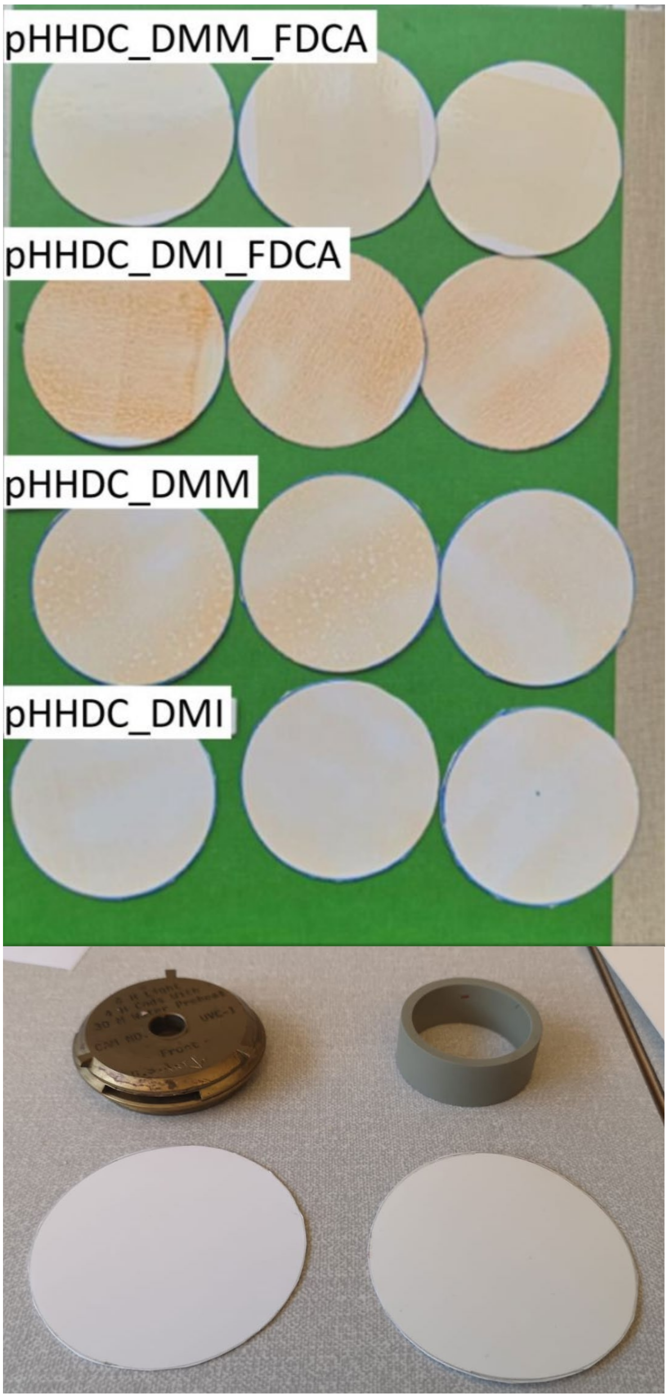

Figure S19. Circular pieces coated with HHDC-based polyester for the Cobb test. Bottom: the modified Cobb-setup: metal weights and rubber cylinder.

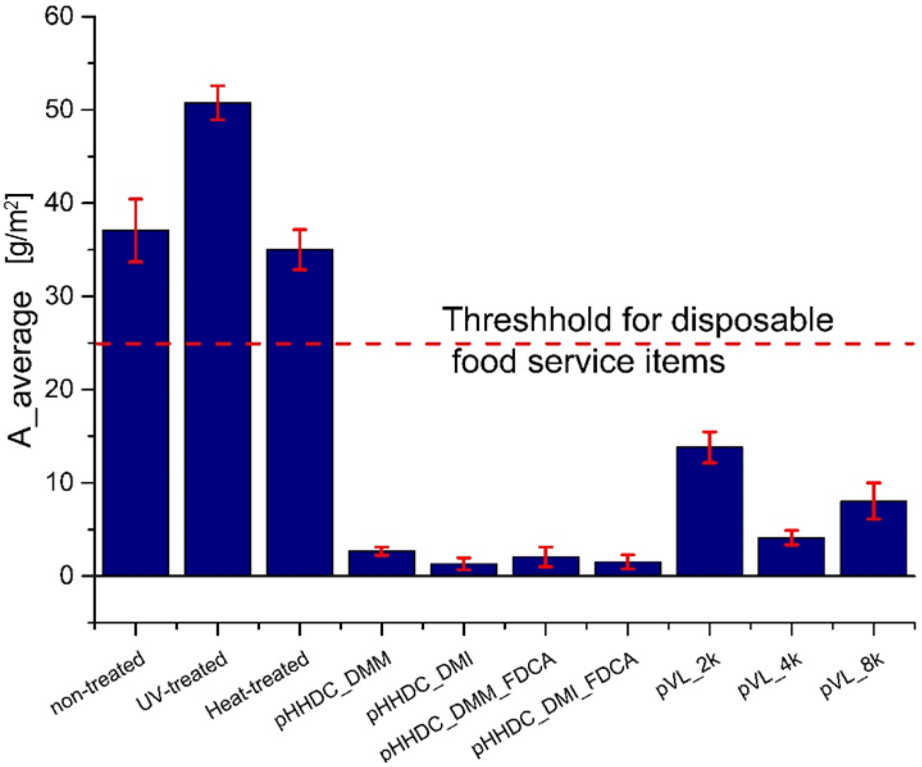

Figure S20. Overview over the performed $Cobb_{120}$ test. Note that for the names of experiments shown, pVL is synonymous to pVaL (scheme 1 in main paper).

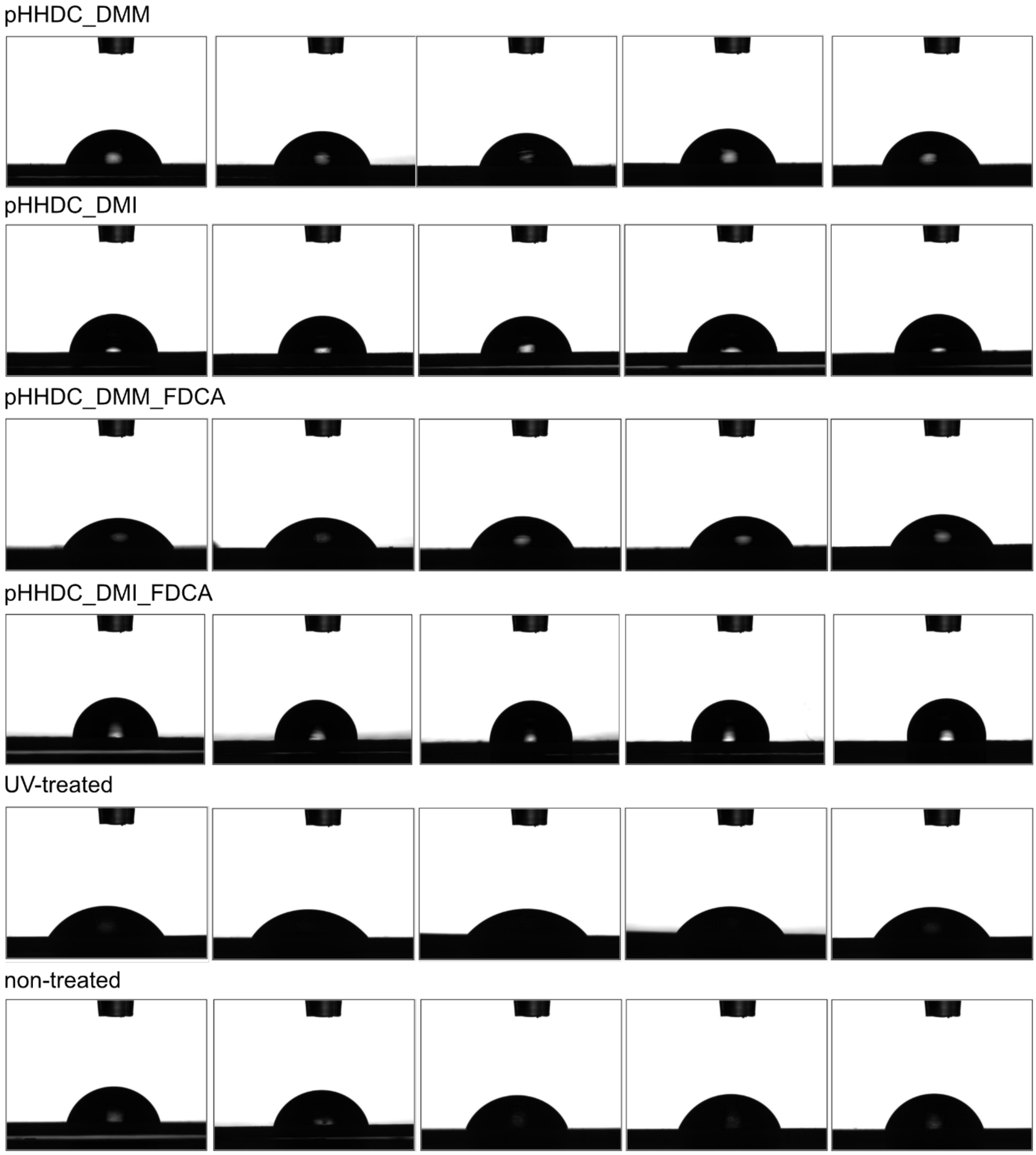

Figure S21. Contact angle (CA) measurements of HHDC based materials. Measurements were conducted for 10 seconds and CA were continuously measured. Average contact angles are reported in Table 2. Pictures were taken at the end of each run.

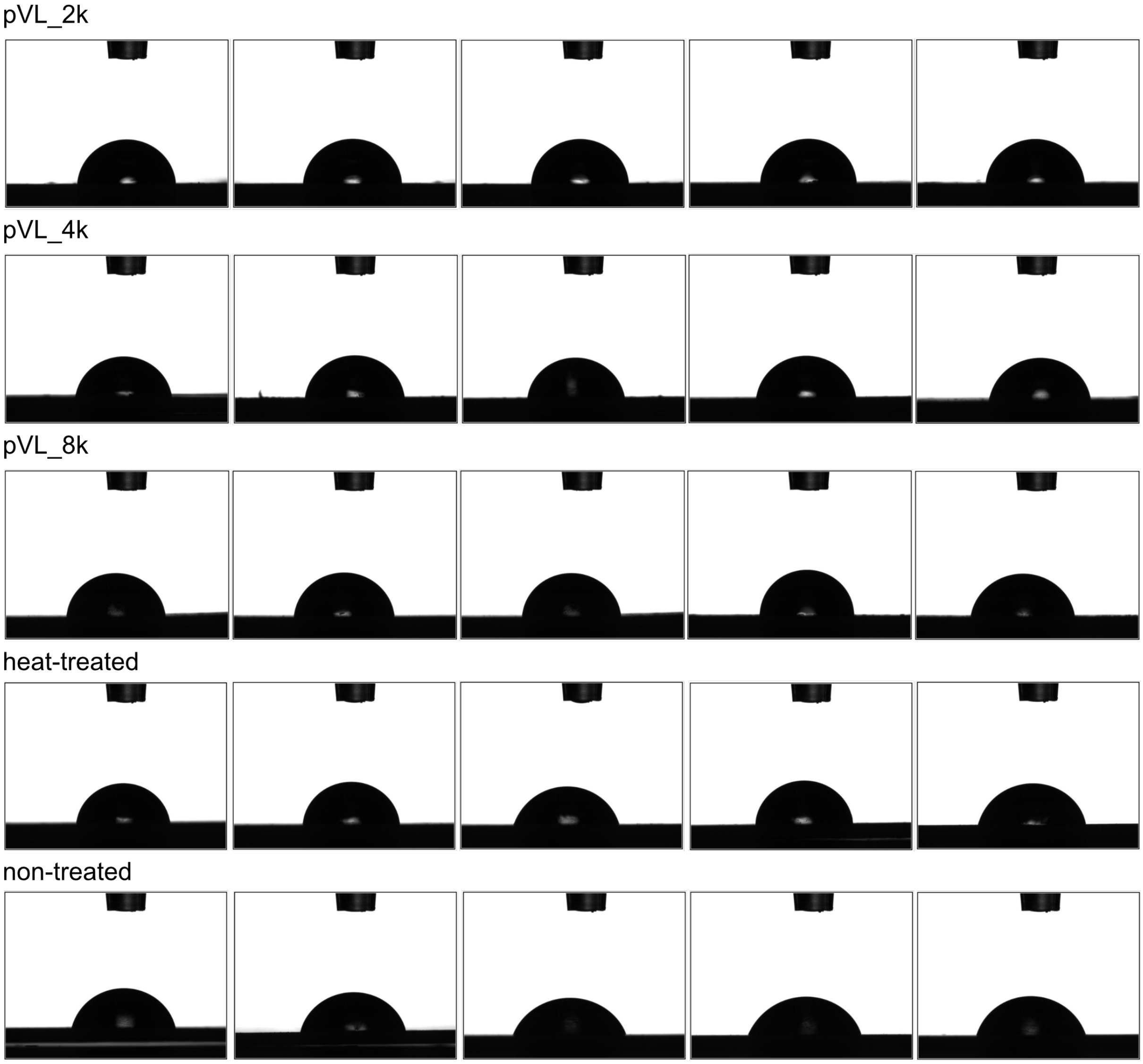

Figure S22. Contact angle measurements of HHDC based materials. Measurements were conducted for 10 seconds and CA were continuously measured. Average contact angles are reported in Table 2. Pictures were taken at the end of each run. Note that for the names of experiments shown, pVL is synonymous to pVaL (scheme 1 in main paper).

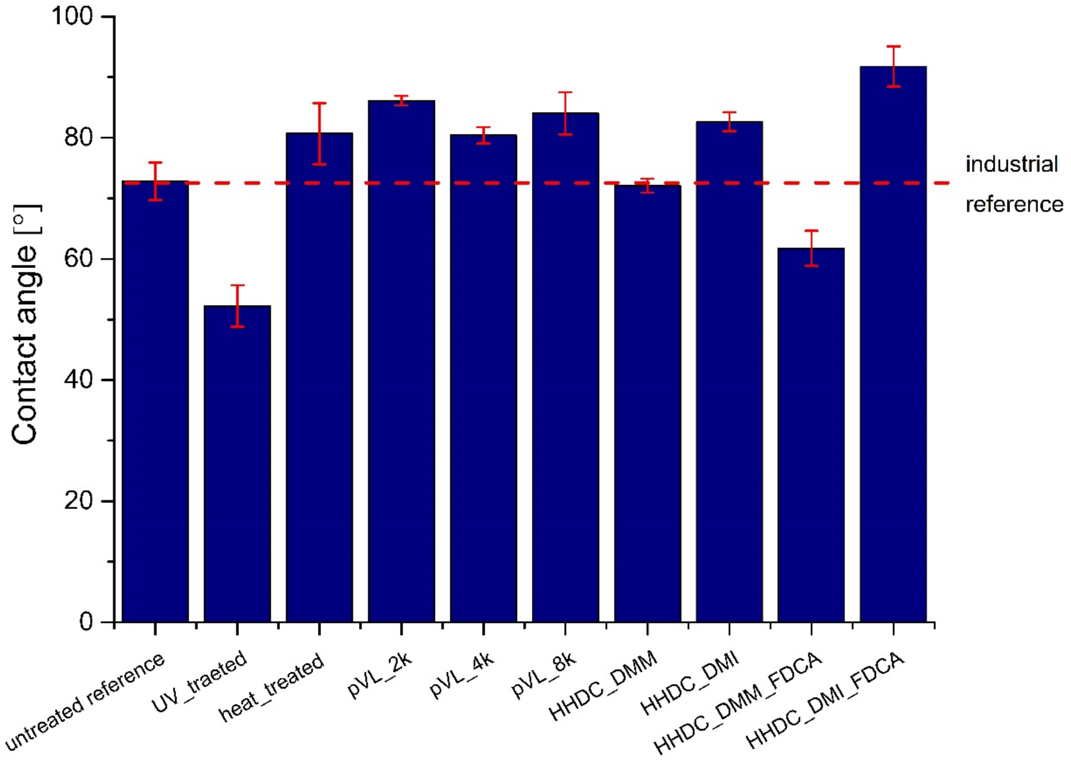

Figure S23. Overview over the performed contact angle analyses. Note that for the names of experiments shown, pVL is synonymous to pVaL (scheme 1 in main paper).

10. Synthesis of sobrerol lactone

Building up on our study on the use of sobrerol as an intermediate building block for the synthesis of functional polymethacrylates and with the aim to explore new paths towards bio-based polyesters, we further investigated the versatility of sobrerol as an intermediate building block. Our strategy was to transform sobrerol into its lactone form, leaving the tertiary alcohol group unreacted during the polymerization and ready for post-functionalization.

Chemical Synthesis of the sobrerol lactone.
The sobrerol lactone (SobL) was synthesized in a three-step reaction. In the first step, *trans*-sobrerol was oxidized using manganese oxide, yielding in carvone hydrate in 80% yield. The double bond in ketone was then quantitatively reduced using hydrogenation chemistry (Fig. S24a and b). Afterwards, 8-hydroxy-*p*-menthan-2-one was oxidized using the same principle as described for verbanone **2** resulting in lactone in 70% yield after flash chromatography, respectively (Fig. S24c). Surprisingly, the produced lactone was not the 7-membered SobL but the respective five membered rearrangement product. The rearrangement was quantitative and occurred in both the chemical and the enzymatic transformation. The 5-membered lactone was identified by the too low shift for the alpha proton of the 7-membered lactone (3.6 ppm), as well as the two peaks originating from the endocyclic methylene groups of the 5-membered lactone (1.45 and 1.3 ppm), which show up as one peak in previous intermediates. To further proof the existence of the 5-membered lactone, the tertiary alcohol was reacted with trifluoroacetic anhydride. The resulting shift of the proton neighbouring the tertiary alcohol and the shift of the exocyclic methylene group clearly identified the 5 membered isomers (Figure S25). To prevent this isomerization from happening, one method could be to protect the tertiary alcohol prior to the Baeyer-Villiger reaction and deprotect again after polymerization. This could potentially lead to a built-in trigger for degradation of the polymer. However, it should be considered that once polymerized, the ring strain of the lactone cannot act as driving force for the rearrangement, which potentially prevents the polymer units from degrading even post deprotection. The 5-membered lactone was attempted to polymerize but results are premature and will therefore be part of future publications.

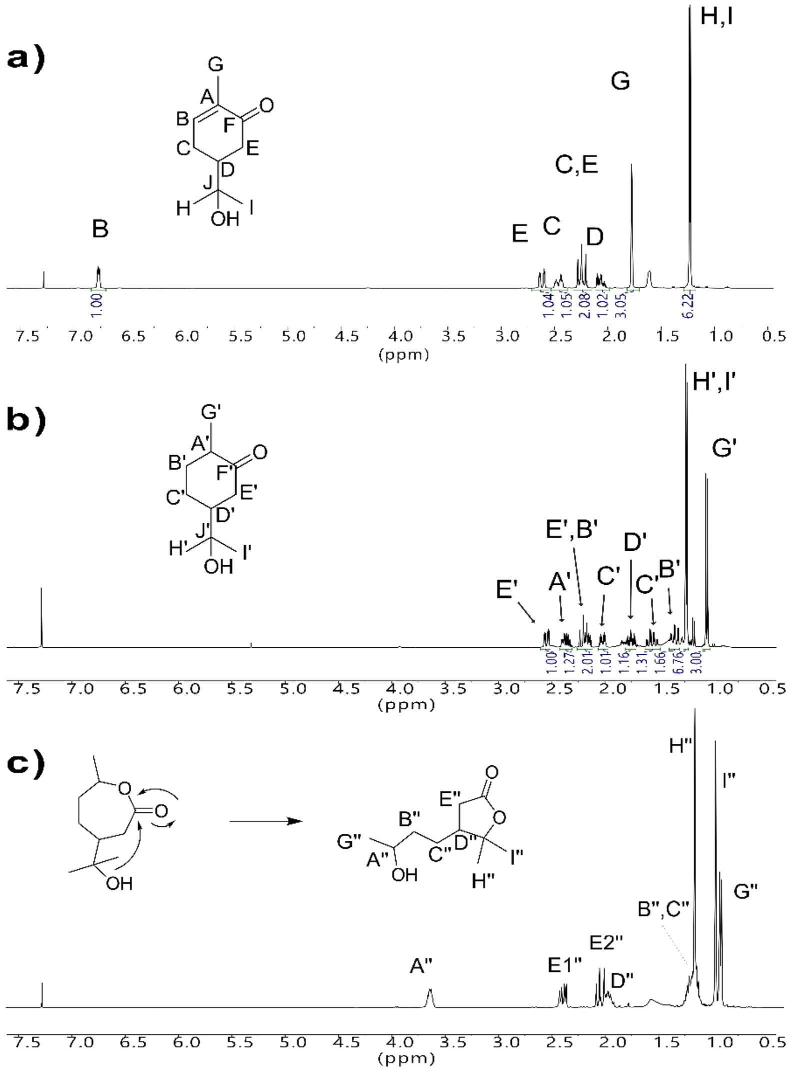

Figure S24. Chemical intermediates during SobL synthesis. Upper spectrum: carvone hydrate, middle spectrum: 8-hydroxy-*p*-menthan-2-one and lower spectrum: sobrerol-based lactone.

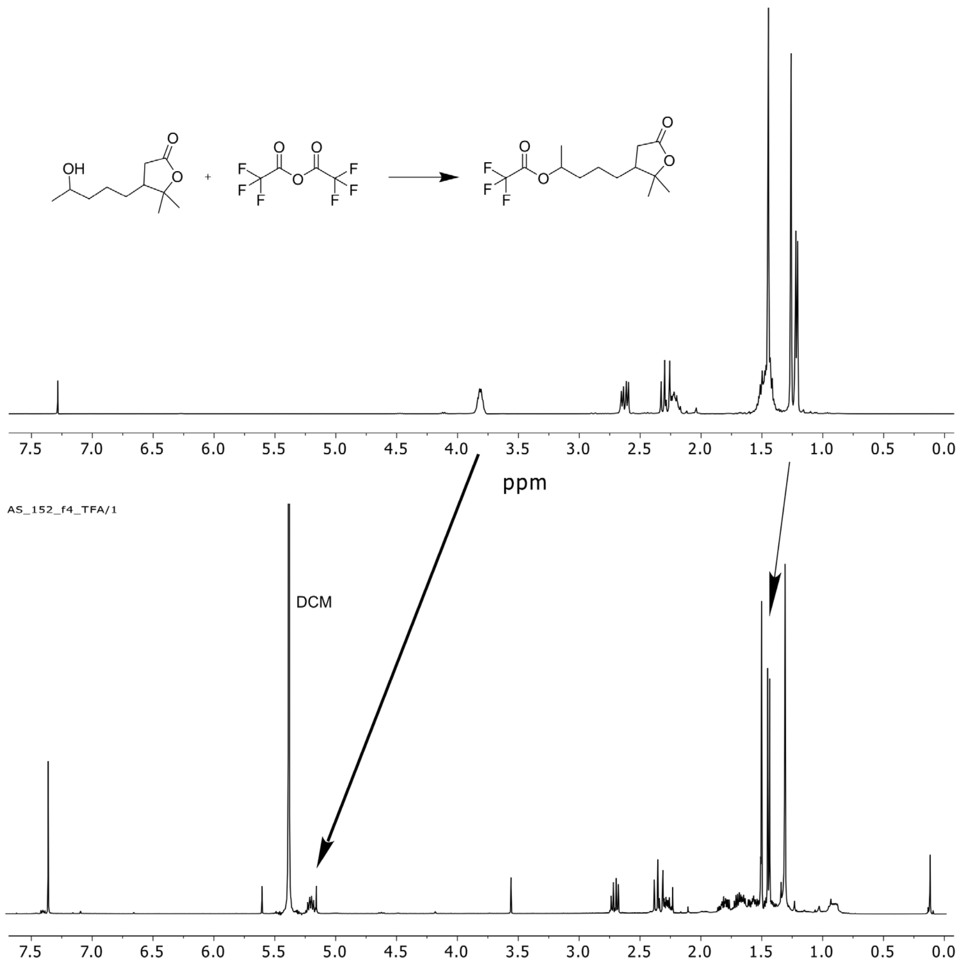

Figure S25. ^{1}H-NMR of SobL (up) and the crude reaction product of the reaction with Trifluoroacetic anhydride. Arrows used to highlight relevant shifts.

Enzymatic synthesis of sobrerol lactone

Expression and extraction of XenB

One *E. coli* colony harboring XenB in plasmid pGAS-XenB-C-His6 was picked to prepare overnight culture in 2YT media with 100 µg/ml ampicillin. The next day, a second culture was prepared from the overnight culture with a start OD600 of 0.1. Once the OD600 reached 0.6, 0.1 mM IPTG (final concentration) was added to induce the expression of XenB that was set for 20 hours at 25 °C 180 rpm. Harvesting of the cells, extraction of XenB, confirmation of expression, and protein concentration assessment was performed in the same way as described for $CHMO_{Actineto}$ in Materials and Methods.

Biotransformation

The biocatalytic conversion of unsaturated ketone derivative of sobrerol **9,** to lactone was carried out in closed 20 ml glass vials, at 30°C and 200 rpm for 24 h. 200 µL fresh cell lysate of both XenB and CHMO_QM_L426A and 4 mM of unsaturated ketone (1 M stock in DMF) were mixed with 62 µM FAD, 0.2 mM NADPH, 0.578 U/µl catalase, 0.2 M filter-sterilized glucose, 0.9 mg/ml glucose dehydrogenase (GDH), supplemented with 50 mM Tris-HCl pH 7.4 to a final volume of 2.5 ml. All ingredient stocks were diluted in 50 mM Tris-HCl pH 8.5, except catalase which was added directly from its vial. GDH and glucose were added as co-factors for NADPH recycling. After 24 hours, the analytes were extracted with ethyl acetate to be analyzed by GC-MS (Shimadzu, GCMS-QP2010 Ultra) on a Rxi®-5ms column (30 m, 0.25 mm [inner diameter], 0.25 µm [film thickness], RESTEK). The temperature program started at 70 °C and increased with 10 °C/min until 230 °C, followed by 15 °C/min to 330 °C where the temperature was fixed for two minutes. The biotransformation was performed two times, in both duplicates the conversion of the unsaturated ketone to the saturated ketone was 100%, and based on peak area 25.9-35.0% was converted into lactone.

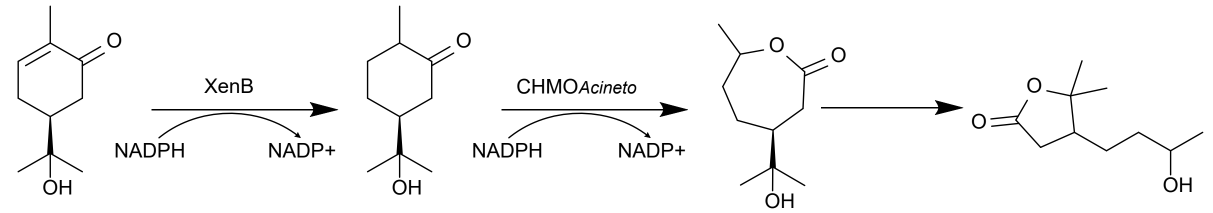

Scheme S1. Biotransformation of unsaturated ketone derivative of sobrerol to lactone. XenB reduces the unsaturated ketone, CHMO*Acineto* (CHMO_QM) performs Baeyer-Villiger oxidation of the saturated ketone, transforming it into the seven-member ring lactone which spontaneously rearranges to its five-member ring structure.

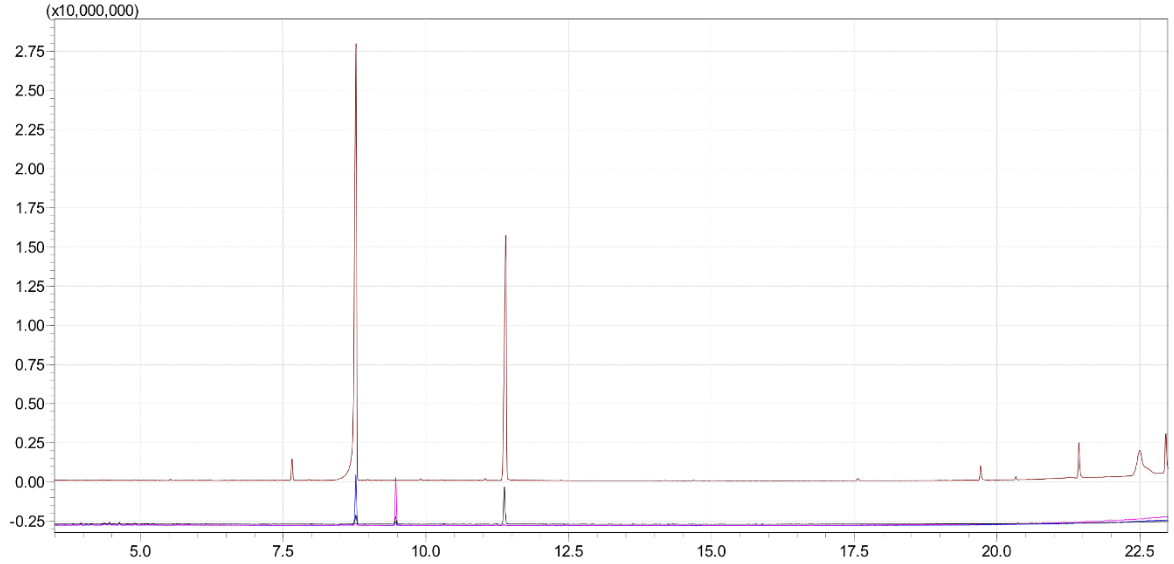

Figure S26. GC-MS chromatogram of extract of biotransformation from unsaturated ketone derivative of sobrerol to five-member ring lactone. At the bottom are the chemically synthesized standards; saturated ketone (8.8 min), unsaturated ketone (9.5 min), and five-member ring lactone (11.4 min). The other peaks were also visible in empty cell control.
